# A Functionally Conserved Gene Regulatory Network Module Governing Olfactory Neuron Diversity

**DOI:** 10.1371/journal.pgen.1005780

**Published:** 2016-01-14

**Authors:** Qingyun Li, Scott Barish, Sumie Okuwa, Abigail Maciejewski, Alicia T. Brandt, Dominik Reinhold, Corbin D. Jones, Pelin Cayirlioglu Volkan

**Affiliations:** 1 Department of Biology, Duke University, Durham, North Carolina, United States of America; 2 Department of Biology, University of North Carolina at Chapel Hill, Chapel Hill, North Carolina, United States of America; 3 Carolina Center for Genome Sciences, University of North Carolina at Chapel Hill, Chapel Hill, North Carolina, United States of America; 4 Department of Mathematics and Computer Science, Clark University, Worcester, Massachusetts, United States of America; 5 Duke Institute for Brain Sciences, Duke University, Durham, North Carolina, United States of America; New York University, UNITED STATES

## Abstract

Sensory neuron diversity is required for organisms to decipher complex environmental cues. In *Drosophila*, the olfactory environment is detected by 50 different olfactory receptor neuron (ORN) classes that are clustered in combinations within distinct sensilla subtypes. Each sensilla subtype houses stereotypically clustered 1–4 ORN identities that arise through asymmetric divisions from a single multipotent sensory organ precursor (SOP). How each class of SOPs acquires a unique differentiation potential that accounts for ORN diversity is unknown. Previously, we reported a critical component of SOP diversification program, Rotund (Rn), increases ORN diversity by generating novel developmental trajectories from existing precursors within each independent sensilla type lineages. Here, we show that Rn, along with BarH1/H2 (Bar), Bric-à-brac (Bab), Apterous (Ap) and Dachshund (Dac), constitutes a transcription factor (TF) network that patterns the developing olfactory tissue. This network was previously shown to pattern the segmentation of the leg, which suggests that this network is functionally conserved. In antennal imaginal discs, precursors with diverse ORN differentiation potentials are selected from concentric rings defined by unique combinations of these TFs along the proximodistal axis of the developing antennal disc. The combinatorial code that demarcates each precursor field is set up by cross-regulatory interactions among different factors within the network. Modifications of this network lead to predictable changes in the diversity of sensilla subtypes and ORN pools. In light of our data, we propose a molecular map that defines each unique SOP fate. Our results highlight the importance of the early prepatterning gene regulatory network as a modulator of SOP and terminally differentiated ORN diversity. Finally, our model illustrates how conserved developmental strategies are used to generate neuronal diversity.

## Author Summary

*Drosophila* uses 50 different olfactory receptor neuron (ORN) classes that are clustered in combinations within distinct sensilla subtypes to decipher a complex chemical environment. Each sensilla subtype houses 1–4 ORN identities that arise through asymmetric divisions from a single multipotent sensory organ precursor (SOP). How each class of SOPs acquires a unique differentiation potential that accounts for ORN diversity is unknown. Here, we show that Rn, along with BarH1/H2 (Bar), Bric-à-brac (Bab), Apterous (Ap) and Dachshund (Dac), is part of a conserved proximodistal (PD) gene regulatory network module that patterns the antennal disc into seven concentric rings and diversifies SOP identities. Each ring expresses a unique combination of the aforementioned transcription factors, and encodes the differentiation potentials for a limited number of sensilla subtypes. Genetic perturbations of the network lead to predictable changes in ORN diversity. These data suggest that the diversification of precursor fields by the prepatterning network is the first step to neuronal diversification, followed by SOP selection by proneural genes, and Notch-mediated neurogenesis. As each step operates in a context-dependent manner, deployment of the same transcription factor network module may regulate neuronal diversity in parallel systems with completely different fate outputs.

## Introduction

Making sense of a complex environment requires a high level of functional diversity in neuronal classes that comprise both the peripheral and central nervous system. Little is known about how limited genetic resources are utilized to reproducibly spawn a large number of neuronal classes. Sensory systems, especially the olfactory system, are prime examples of both this neuronal diversity and how it enables organisms to survive in a complex world. The olfactory system drives behaviors fundamental to organisms’ survival, like foraging, toxin and predetor avoidance, as well as social behaviors such as courtship, aggression and parenting [1]. To detect and decifer the chemical cues shaping these behaviors, animals are equipped with a diverse array of olfactory receptors (ORs) that evolve rapidly [2–6].

The *Drosophila* olfactory system is a great model to study neuronal diversification because: (1) the organizational principle of the olfactory system is conserved across species; (2) it is a complex system with sufficient diversity that calls for sophisticated mechanisms of differentiation; yet, (3) its numerical complexity is much reduced as compared to mammals, which makes systems-level investigation possible. Adult flies have two pairs of olfactory sensory appendages: the third segment of antenna (funiculus) and the maxillary palp [7]. The surfaces of these olfactory organs are covered by multiporous sensory hairs, called “sensilla”. Each antenna and maxillary palp contains about 410 and 60 sensilla, respectively, that house clusters of 1–4 olfactory receptor neurons (ORNs) [8,9]. There are approximately 1300 ORNs per antenna and 130 per maxillary palp [8,10]. Each ORN typically expresses a single receptor gene from a repertoire of 80 genes, creating a total of 50 adult ORN classes that are clustered into stereotypical combinations within 22 individual sensilla subtypes [11].

Antennal sensilla have three major morphological types: club-shaped basiconica (ab: antennal basiconic), spine-shaped trichoidea (at), and cone-shaped coeloconica (ac), in addition to the rare intermediate type (ai) [10]. Basiconic sensilla are subdivided into large, thin and small types. Each morphologically distinct sensilla type is further segmented into generally 4 or 3 sensilla subtypes, which are defined by the unique subsets of ORN classes that express invariable combinations of olfactory receptors [7,9,12]. Basiconic and trichoid sensilla contain ORNs that express conventional insect OR genes, except for two ORN classes (Gr21a/Gr63a- and Or10a/Gr10a-expressing neurons) in the large basiconic subtype ab1 that (co-)express gustatory receptors (GRs) [13,14]. Coeloconic sensilla generally contain ionotropic receptor (IR)-expressing ORNs [15–17]. Because of the zonal localization of sensilla types/subtypes and their defined relationships to olfactory receptor genes, the expression of a given receptor is accordingly restricted to a specific zone, and thus all ORNs collectively form a sensory map on the antenna [7,18–20]. Interestingly, despite the evolutionary separation between *Drosophila* and mammals, the principle of zonal restriction of OR expression seems to be conserved [21–23]. It is unclear, however, how different zones are generated and how they regulate the distribution and diversity of different ORN classes.

In flies, the olfactory appendages develop from the antennal discs, which are specified by Distal-less (Dll), Homothorax (Hth) and Extradenticle (Exd) [24,25]. *hth* is an anterior-posterior (A/P) homeotic selector gene that is sufficient to confer antennal identity in other tissues. Likewise, the homeotic gene *antennapedia* (*antp*) induces leg fate and a gustatory appendage [25–29]. The legs contain gustatory receptor neurons (GRNs) that sense non-volatile chemicals, and GRNs also display neuronal diversity with distinct receptor profiles [30–32]. Both the antennae and legs are ventral, segmented appendages and parallels between them have been drawn for years [26–29]. Indeed forced expression of *antp* can transform antennae into legs [26–28]. In either case, the proximodistal (PD) axis of the 3D adult tissue is constructed by the extension of the 2D sheet-like imaginal disc from the center. True joints are formed along this axis in both appendages, although the legs are more segmented [11,33,34]. In addition, the alignment between segments appears to be more linear in the leg, reflecting the “telescope-out” motion of the disc during the morphogenic event as opposed to the “fanning” motion in the antenna. Both sensilla-covered chemosensory organs (funiculus and tarsi) develop from the distal regions of the corresponding discs. The tarsi are further segmented, which sets natural boundaries for the position of a given GRN class. In contrast, the funiculus possesses a contiguous anatomy allowing the flow of ORN precursors within a certain range [11,33,35]. It is believed that fly antennae and legs are evolutionarily related, and some common molecules have been discovered to account for the segmental features of their tissue-level analogy along the PD axis [27,28,36,37]. However, how the differentiation of the cellular components, especially the complex array of chemosensory neurons housed in the antennae/legs, is coordinated with or by these morphogenic events remains a mystery.

We recently reported that the *rotund (rn)* gene locus, known to control tarsal segmentation, has a critical function in diversifying ORN classes during the antennal disc development [38,39]. Rn is required in a subset of sensory organ precursors (SOPs) to confer novel sensilla subtype differentiation potentials from some default potentials within each sensilla type lineage. In *rn* mutants, ORNs in *rn*-positive sensilla subtype SOPs are converted to lineage-specific default *rn*-negative fates, resulting in only half of the normal ORN diversity. Through a developmental transcriptome analysis and in light of the knowledge about leg development, we found that Rn, together with BarH1/H2 (B-H1/2, Bar or B), Apterous (Ap), Dachshund (Dac), and Bric-à-brac (Bab), is part of the conserved PD gene regulatory network module that plays a crucial role in patterning the antennal precursor field prior to proneural gene-mediated SOP selection. Interactions among these PD genes separate the developing antennal disc into seven concentric domains. Each ring is represented by a unique combination of the aforementioned transcription factors, and encodes the differentiation potentials for a limited number of sensilla subtypes. Genetic perturbations of the network lead to predictable changes in the ratios of different sensilla subtypes and corresponding ORN classes. In addition, using endogenously tagged Rn protein *in vivo*, we show direct binding of Rn to *Bar* and *bab* regulatory regions in the antennal disc. This same network module was previously shown to control the segmentation of tarsi in the developing leg and we show that Rn controls neuronal development of the GRNs in the leg as well. We propose a three-step mechanism to explain ORN diversification, beginning with the prepatterning of the precursor field by a gene regulatory network, followed by SOP selection by proneural genes, and Notch-mediated neurogenesis leading to terminal differentiation. The final precursor potentials are largely determined by the prepatterning phase. In our model, each step operates in a context-dependent manner: in a different context, the same transcription factor network with the same logic steps can result in completely different neuronal identity outputs. This combinatorial approach enables the same small, conserved set of genes to specify, in parallel, a broad range of chemosensory neurons.

## Results

### A time-course RNAseq analysis reveals mis-regulation of developmentally critical genes in *rn* mutant

Previously, we demonstrated that the Krüppel-like transcription factor Rn cell-autonomously diversifies ORN classes by branching off novel sensilla subtype lineages from parallel default ones. In *rn* mutants, ORN diversity is reduced almost by half. Neurons from at4 sensilla in the trichoid zone, ac2 in the coeloconic zone, and ab1 and ab9 in the basiconic zone are all expanded at the expense of specific ORNs in *rn*-positive sensilla subtypes [38]. To reveal the molecular mechanism by which Rn modulates ORN precursor identities, we compared transcript abundances from a time-course RNAseq analysis in wild type (*w*^*1118*^), heterozygous and homozygous *rn* mutant flies (see Materials and Methods) at four temporal landmarks during antennal development. We surveyed the prepatterning (larval), SOP selection (8hr pupal), neurogenesis (40hr pupal) and terminally differentiated adult stages [38,40–44]. For the adult stage, changes in OR expression in *rn* mutants were consistent with the overall trend described from our previous report (S2 Fig), suggesting that our experiment effectively identifies genes whose expression is affected by *rn*.

To find key developmental genes likely acting downstream of Rn, we focused on the three early stages. As Rn is only expressed during larval and early pupal periods, we reasoned that the genes under direct Rn control would show differential expression in one or more of these early time points. A Venn diagram generated from the final lists for all early stages reveals that some genes may be misregulated only in one particular stage, while others show misregulation—both up and down—across multiple stages (S1A and S1B Fig, also see Materials and Methods).

**Fig 1:**
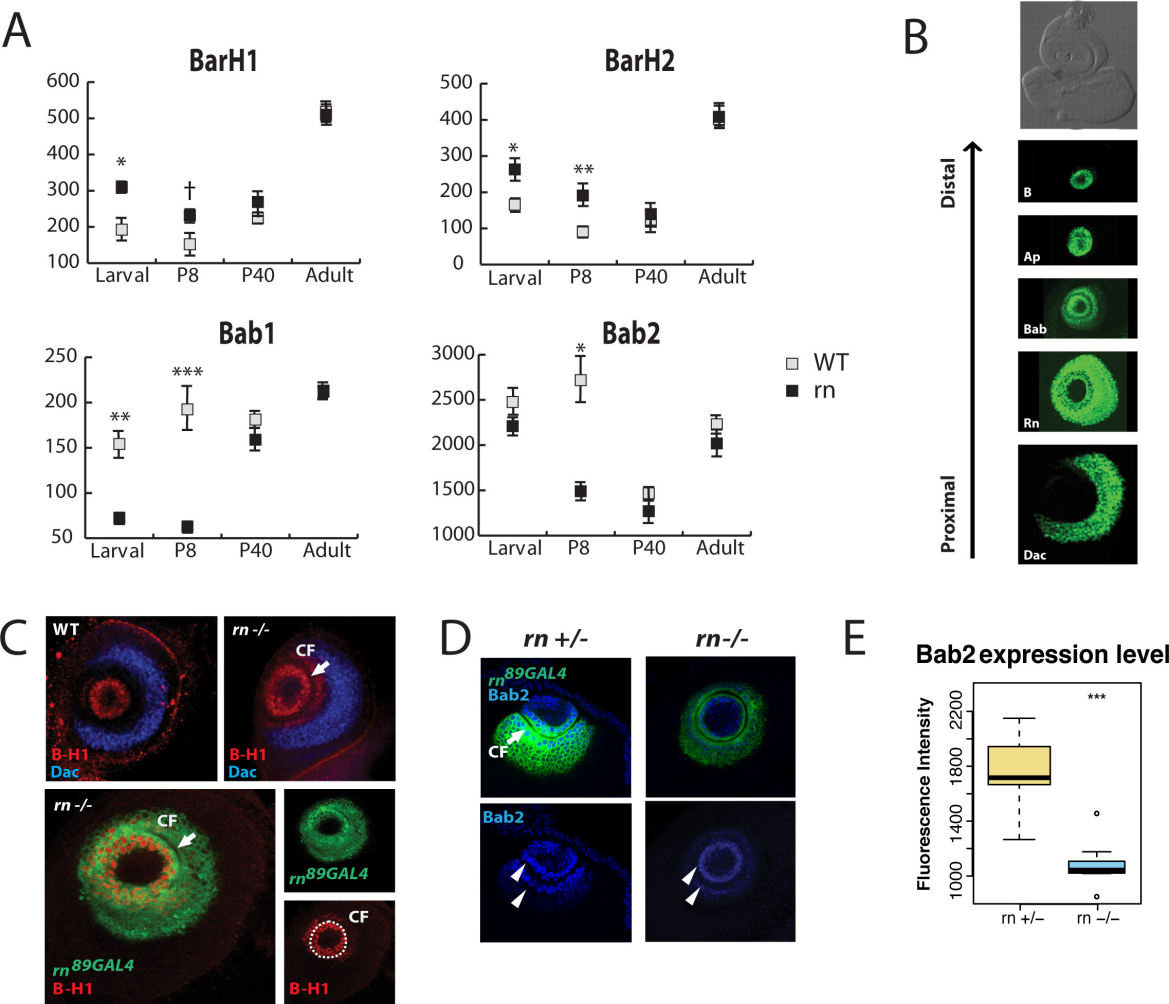
Rn controls the expression of *BarH1/2* and *bab1/2* in the antennal disc. (A) Expression levels of *B-H1/2* and *bab1/2* between the control and mutants in different stages. Normalized expression data by DESeq2 was used. † *p*<0.1 * *p*<0.05 ** *p*<0.01 *** *p*<0.001. (B) Expression patterns of PD genes in the third instar larval antennal disc. They are expressed in concentric rings along proximodistal axis. Anti-B-H1 (T. Kojima), anti-Bab2 (F. Laski), anti-Dac were used to visualize Bar, Bab and Dac. *ap*^*rK568*^ (stained with anti-β-gal) and CRISPR tagged Rn-EGFP [49] were used to visualize Ap and Rn. The antennal disc is the upper portion of the eye-antennal disc complex in the bright-field image. Images were taken from different animals. (C) Confirmation of RNAseq results on Bar expression. Antibodies to B-H1 (red) were used. Expansion of B-H1 outside CF (arrow) is apparent in *rn* mutants. The expansion of Bar is restricted within the distal boundary of Dac (blue). In the expanded zone outside the central fold, Bar expression overlaps with *rn* reporter expression (lower panel). (D) Bab2 antibody staining (blue) shows concentration gradients in wild type. In these composite images, two circles (arrow heads) on the ridges of the central fold show the highest concentrations, but the high level of expression is continuous along the central fold (see S3C Fig). Bab2 level is weaker in *rn* mutants, although the overall pattern is unchanged. The *rn*^*89GAL4*^ reporter labels cells that have active *rn* promoter (green). CF, central fold (arrow). (E) Quantification of Bab2 levels in (F). n = 10, *** *p*<0.001.

GO term analysis showed an excess of misregulated genes with potential functions in development, such as transcription factors and signaling molecules (an in-depth analysis of the dataset is beyond the scope of this study, and will be published elsewhere). In addition, functional clustering analysis using the online tool, DAVID [45,46], for each category in the Venn diagram, uncovered a functional group including homeodomain(-like) proteins BarH1/2 (B-H1/2, Bar or B) and Bric-à-brac1 (Bab1) as being modified in *rn* mutants. Interestingly, both *B-H1/2* and *bab1* showed changes in transcription levels only during early developmental stages (Fig 1A). It is important to note that B-H1 and B-H2, as well as Bab1 and Bab2, are functionally redundant, and have extensively overlapping expression patterns (S3B Fig) [47,48]. Because only *bab1* but not *bab2* was included in the initial functional clustering analysis, we re-examined the RNAseq datasets for *bab2*. We found that *bab2* had an overall higher level of expression than *bab1*, and similar trend of misregulation exists for *bab2* (Fig 1A). While the *p* values were still above the arbitrary cutoffs in several cases—likely due to the cellular heterogeneity of antennal disc samples used in transcriptome analysis—the interrelatedness of these genes and *rn* suggested that they together might have an important role in ORN diversity. Thus, we focused on B-H1/2 and Bab1/2 in this study and explored their roles in ORN diversification further.

### A common molecular network patterning the antennal and leg appendages

Rn was previously reported to function in a gene regulatory network together with B-H1/2, Dachshund (Dac), Apterous (Ap) and Bab1/2 to pattern the segmentation of the *Drosophila* leg disc in the proximodistal (PD) axis [49,50]. In the leg, the temporally dynamic PD gene regulatory network, under the influence of morphogen gradients, defines a number of concentric domains on the leg disc via cross-regulation, which in turn determines individual segment identities. These data led us to hypothesize that the neuronal diversity phenotypes observed in *rn* mutants arise due to the changes of expression domains for the PD network components during antennal disc patterning. To test this hypothesis, we first systematically examined the spatial patterns of these factors in the developing antennae of wild type animals, and found that each factor is expressed in a concentric ring along the PD axis of the discs (Fig 1B). The gene expression patterns are remarkably similar between the legs and antennae, suggesting that these two organs share the same molecular tool kits that pattern their respective discs [39,51–53].

Rn was previously shown to be a positive regulator of Bab1/2 and a negative regulator of B-H1/2 in the developing leg disc [39,51]. Given the evolutionary relationship between the leg and the antennae, we thought a similar regulatory network may exist in the antennal disc [54]. Indeed, the regulatory relationships of PD genes from the leg-patterning network can explain the misregulation of *B-H1/2* and *bab1/2* in the antennal disc from our RNAseq data. This idea was then confirmed by examining their *in vivo* expression patterns (Fig 1C–1E and S3A Fig). B-H1 is normally expressed in the center of the disc, bounded by the central fold (Fig 1C). In *rn* mutants, B-H1 is expanded outside of the central cells into cells that are normally *rn*-positive and B-H1/2-negative, but the expansion is confined within the distal boundary of Dac (Fig 1C). The ectopic cells that are labeled with B-H1 antibody in *rn* mutants are positive for the *rn* promoter reporter (Fig 1C), suggesting that this *rn*-positive precursor domain may have switched fates as a result of the loss of Rn and the expansion of B-H1/2. On the other hand, Bab2 expression is significantly reduced in *rn* mutants (Fig 1D and 1E). Consistent with the RNAseq results, we did not detect obvious changes in *ap* expression in the third instar larval stage (S3A Fig). Taken together, these results suggest that a common PD gene regulatory network module operates in parallel during leg and antennal disc development.

### Expansion of Bar expression in *rn* mutants underlies expansion of a default fate and changes in ORN diversity

**Fig 2:**
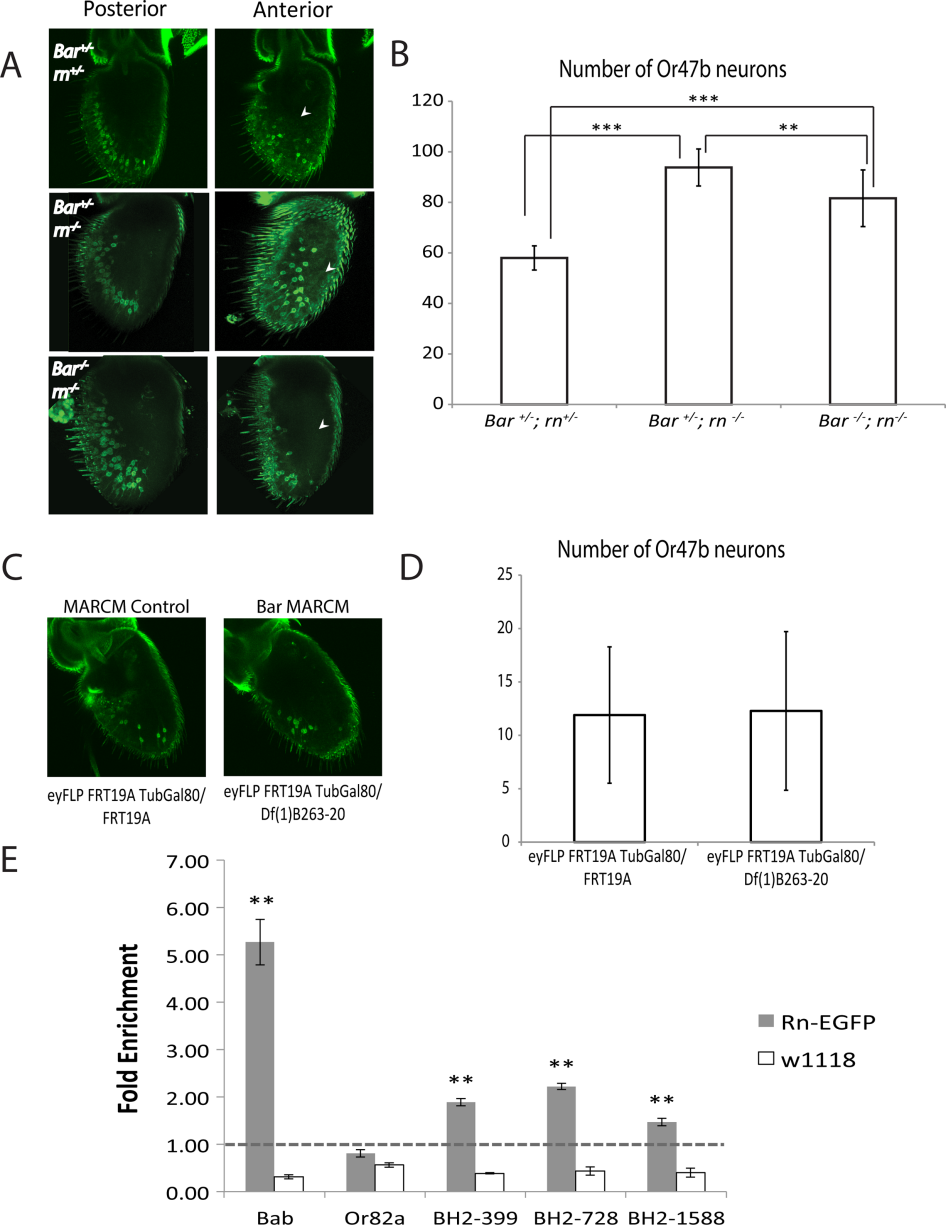
Relationship between *BarH1/2* and *rn*. (A) Expression of Or47b from at4 sensilla as detected by Or47b::mCD8-GFP reporter in control, *rn* single mutant and *rn*/*Bar* double mutant flies. Or47b expression is expanded into an anterior zone (right panel, arrowhead) in the *rn*-/- antenna. When mutant clones for *BarH1/2* have been introduced in the *rn*-/- background, the expansion of Or47b expression in the anterior zone is no longer obvious (arrowhead). (B) Quantification of the numbers of Or47b expressing neurons from (A). There is a significant decrease in the number of Or47b neurons in *Bar/rn* double mutants as compared to *rn* single mutants. ANOVA revealed a statistically significant change in the number of Or47b neurons (p<0.001, Fcrit = 3.25, df = 39). Statistics displayed represent post-hoc Student’s T-tests. n = 15. **p<0.01, ***p<0.001. (C) Or47b neurons in wildtype and *Bar* MARCM clones. Or47b neurons were labeled using Or47b-GAL4 to drive UAS-CD8 GFP. Clones were generated using ey-FLP FRT19A TubGAL80. (D) There was no detectable difference between the numbers of Or47b neurons in wildtype and mutant clones. Quantification showed that the average number of cells observed was approximately one fifth of the total number of Or47b neurons in (B), suggesting that MARCM occurred in a small portion (20%) of the antennal disc. (E) ChIP-qPCR using anti-EGFP antibodies to pull down endogenous Rn-EGFP from third instar antennal discs and test the binding of Rn to *Bar*/*bab* regulatory regions. T13 enhancer sequence upstream of *bab2*, which was previously reported to bind to Rn *in vitro*, showed enrichment for Rn-GFP in the ChIP assay. Primers spanning different regions of BarH2 upstream its TSS (BarH2-399, BarH2-728, and BarH2-1588) also showed binding to Rn-EGFP. Or82a, which is expressed in ORNs that develop from *rn-*positive ORNs did not show binding as previously reported [38]. **p<0.01.

In *rn* mutant antennae, the number of ORNs in some *rn-*negative sensilla (e.g. Or47b ORNs in at4) are increased at the expense of ORNs in *rn-*positive sensilla [38], and this occurs in parallel to the expansion of Bar in the antennal disc. To test if the expansion of Bar leads to an increase in at4 ORNs in *rn* mutants, we analyzed *Bar/rn* double mutants. Normally, approximately 60 Or47b ORNs are found in wild type flies, and this number is increased to ~90 in *rn* mutants [38]. We first generated eyFLP-induced MARCM clones, which induced small clones that are either wild type or *Bar* mutant in approximately 20% of all ORNs (Fig 2C and 2D). These analysis showed that the number of total Or47b ORNs in *Bar* mutant clones was not significantly different than that of the wild type clones (Fig 2C and 2D). However, when generating similar *Bar* mutant antennal clones in *rn* mutant animals, we detected a statistically significant suppression of Or47b ORN expansion down to ~80 cells using ANOVA and post-hoc Student’s T-test (Fig 2B, S1 Table, p<0.001, Fcrit = 3.25, df = 39). We specifically detected a loss of Or47b ORNs from the ectopic antennal zone seen in *rn* mutants (Fig 2A). These data suggest that the expansion of Bar is causal for the increase of at4 ORN fates in *rn* mutants. As at4 sensilla are *rn* and *dac*-negative [35,38], they are likely developed from the Bar-positive inner circle of the disc (Fig 1B). Consistently, at4 ORNs express the *Bar* promoter reporter (see below for fate mapping and genetic analyses). Remarkably, Bar seems to be dispensable for the endogenous at4 fate (Fig 2C and 2D), presumably due to the robustness of this fate to genetic perturbations.

### Rn directly binds upstream of *BarH2* and *bab2* gene regions *in vivo*

Next we wanted to know if the genes in the network directly regulate each other. We focused on the function of Rn, as this may help explain the misregulation of *B-H1/2* and *bab1/2* in *rn* mutant. Previous *in vitro* assays have shown that Rn binds to a T-rich motif (T13) in the LAE (leg and antennal enhancer) sequence upstream of *bab2* to activate its expression during leg and antennal development. However, *in vivo* evidence for Rn binding targets has been missing due to the lack of a high-quality antibody. We generated a fly line that carries an EGFP endogenous tag for Rn (Rn-EGFP), which was confirmed and validated for functionality [55]. We then used EGFP antibodies to do chromatin immunoprecipitation (ChIP) followed by qPCR to test binding of Rn to *bab* or *Bar* regulatory elements in the antennal discs.

qPCR primers were designed in the first 2kb upstream of the transcription start site (TSS) in the *Bar* loci (Materials and Methods). A primer set covering the T13 motif in the *bab2* enhancer was used as a positive control, while the M1 motif region from Or82a promoter was used as a negative control [38,39]. ChIP on antennal disc tissues was able to detect direct binding of Rn to the published *bab2* enhancer and the promoter region of *B-H2* using the Rn-EGFP line, and further confirms that Rn does not bind to OR promoters (Fig 2E). We noticed that the binding of Rn to *bab2* enhancer is more robust compared to *B-H2* sites, which might arise due to the differences in the genomic organization of these binding sites. Since both *B-H2* and *Or82a* contain T13-like motifs in their upstream regions, the binding of Rn seems to require some special chromatin environment and/or the facilitation of binding by other factors in addition to the presence of a T13 consensus sequence. While our analysis cannot distinguish whether Rn binds to a different motif in the *B-H2* promoter, these results suggest that the concentric TF domains may be formed by cross-regulatory relationships, and that Rn regulates components of the network through directly binding to their regulatory elements.

### Partitioning of prepatterning domains

**Fig 3:**
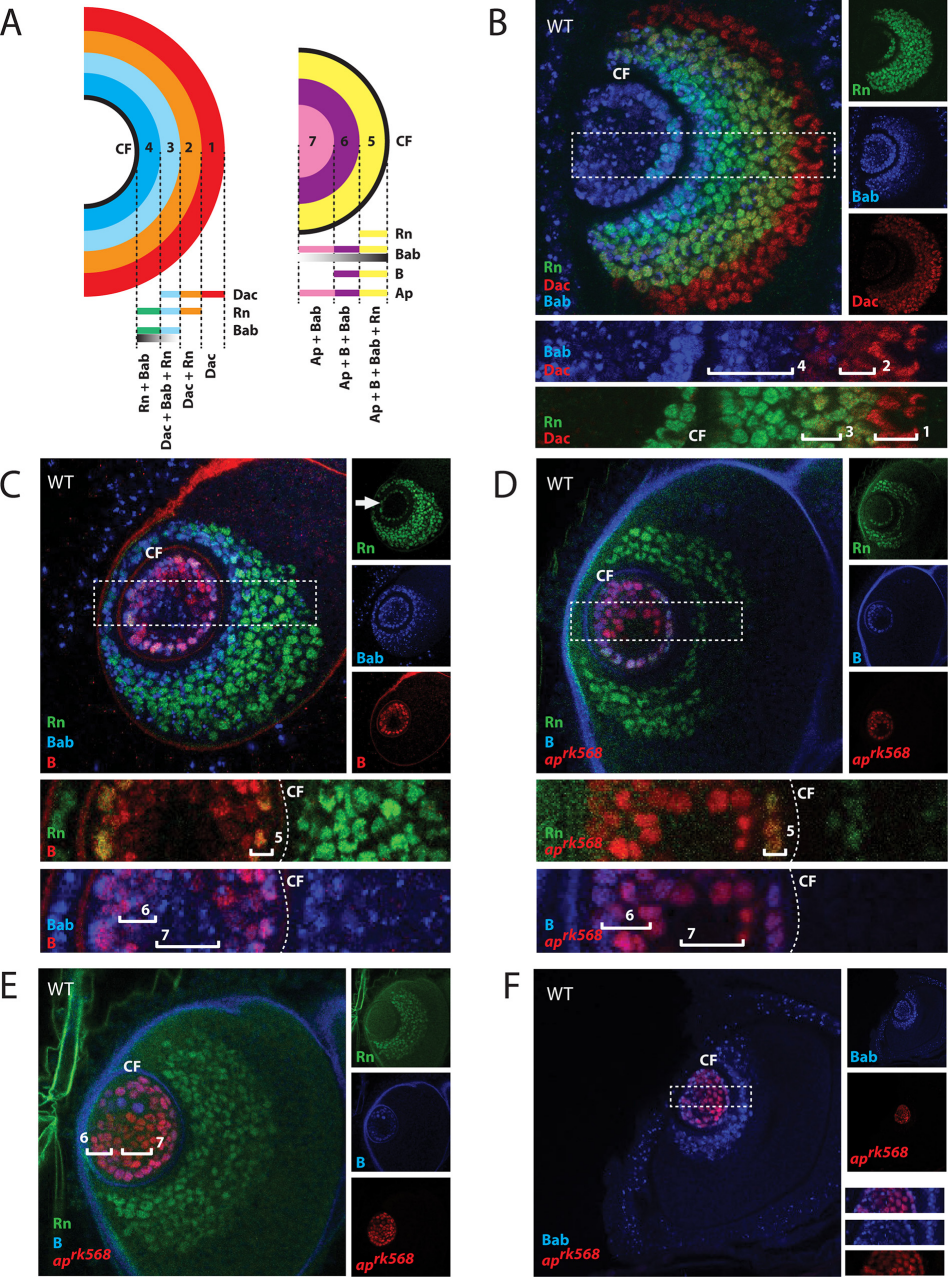
Intercalation of prepatterning genes inside and outside the central fold. (A) A schematic model showing 7 concentric domains in the antennal disc determined by unique combinations of transcription factors. Four rings are placed outside the central fold (CF), and three are inside. (B-F) Expression analyses of Rn, Bab and Dac (B); Rn, Bab, and Bar (C); Rn, Bar and *ap* (D and E); Bab and *ap* (F) demarcate 7 different rings in the antennal discs based on combinations of these factors as well as Bab concentration gradient. Middle confocal sections were shown for (B-D), and superficial sections were shown for (E) and (F). Bab expression is highest near the central fold and decreases towards outermost and innermost regions. Bab was labeled with anti-Bab2. Rn was labeled with tagged EGFP in Rn-EGFP. Dac was labeled with a monoclonal anti-Dac antibody. Bar was labeled with anti-B-H1. *ap* was labeled with anti-β-gal in *ap*^*rK568*^. Individual channels are shown on the right of each image. Boxed areas are shown below in higher magnification. Rn expression is seen as a circle (arrow) inside the central fold in the middle sections (C). Central fold is denoted by a dashed line. Each ring is numbered and marked by a bracket.

We noticed that the expression domains of several PD factors overlap in the third instar antennal disc, and therefore we wanted to dissect the spatial relationships between these factors more carefully. To simplify the descriptions, we use the central fold (CF) as a landmark, which is usually observed as an unstained dark circle in a superficial section of confocal images, to separate the disc into inner and outer regions (Fig 3B and S3C Fig). In the outer region, Dac, Rn and Bab are expressed from more proximal to more distal area in the disc (Fig 3B). Due to the substantial overlap in their expression patterns, these three factors divide the region into four concentric rings. We number the rings starting with the outermost one being R(1), and therefore R(1) to R(4) are assigned to this region (Fig 3A and 3B and S3D Fig). Bab here is expressed in a gradient, similar to its previously reported expression in the leg discs [56]. Our results show that the highest level of Bab is found near the central fold, and its expression decreases toward both outermost and innermost areas of the disc (Fig 3B and S3C Fig).

Three more rings can be found inside the central fold. R(5) is the only ring that shows quadruple labeling by 4 factors examined (Rn, Bab, Ap, and Bar) (Fig 3C and 3D). This ring also corresponds to the only region that expresses Rn inside of the central fold (Fig 3C–3E). Bar expression cannot be detected in the centermost region (Fig 3C–3E). Taken together, the partial overlapping patterns of Dac, Rn, Bab1/2, B-H1/2 and Ap expression demarcate seven concentric ring domains in the third instar antennal disc, and each ring is marked by a unique combination of prepatterning factors (Fig 3A and S3D Fig).

### Sensilla subtype fate mapping onto the prepatterned domains

**Fig 4:**
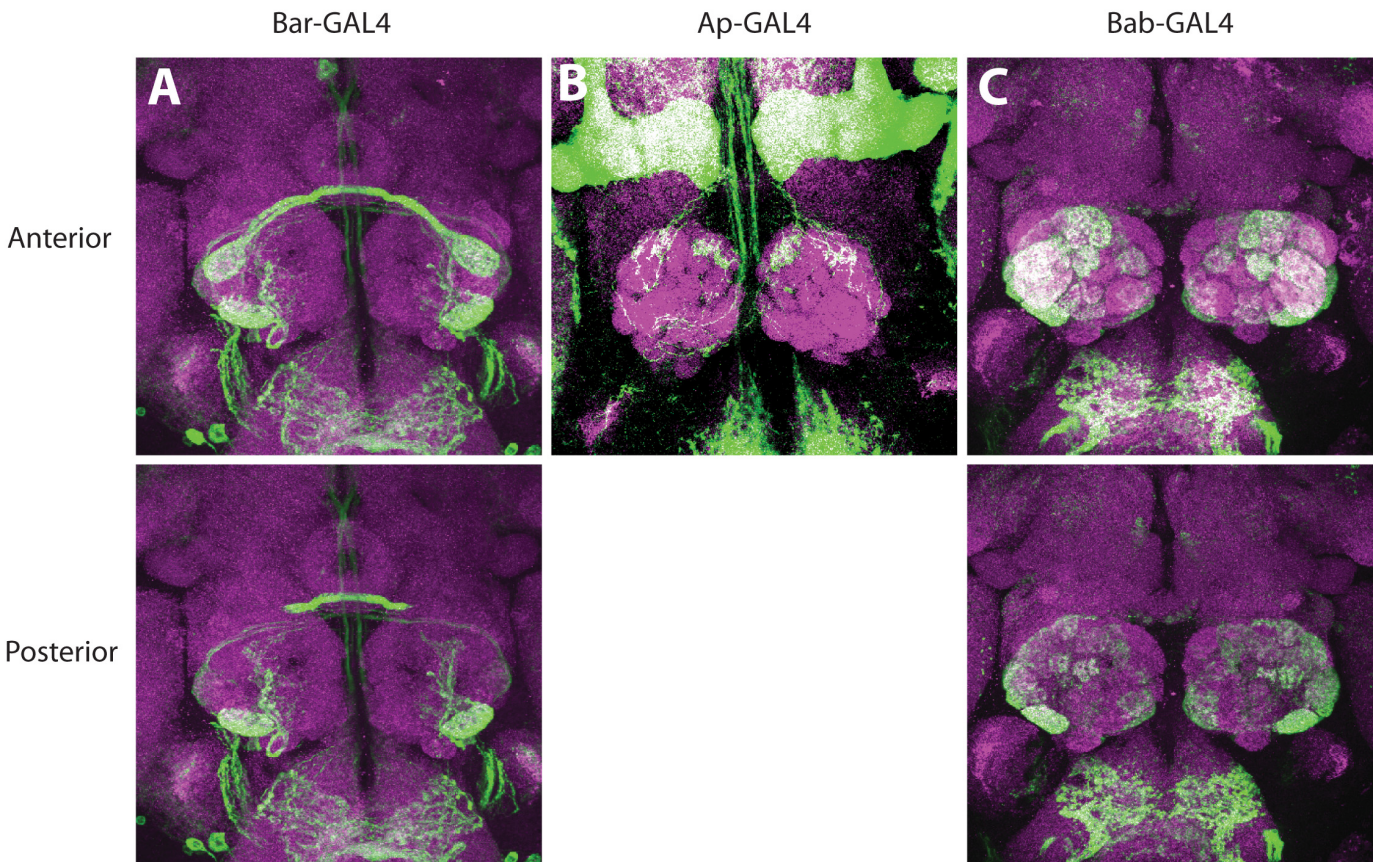
Expression pattern of PD network components in ORNs. Antennal lobe innervation of ORNs expressing GFP driven by *Bar*^*NP4099*^ (A), *ap*^*md544*^ (B), or *bab1*^*Pgal4-2*^ (C). For *bab* and *Bar* expression, analysis was done on adults. For *ap*, 50–75 hour APF pupal brains were examined. Top rows show anterior confocal slices and the bottom rows show the posterior slices. Also see S4 Fig for additional analysis and Table 1 for summary of the results.

Next we asked which precursor identities are generated from each of these seven domains. As all of the components within a sensillum arise from a single SOP, we wanted to know the sensilla subtype identities of SOPs from each concentric domain. To do this, we used promoter-driven reporter lines for each individual gene to label ORN axons. Because ORN sensory identities are closely linked with the glomerular identities in the brain, we can infer which ORN classes express the given factor from the glomerular labeling pattern in this analysis (Fig 4 and Table 1 and S4 Fig). *Bar-* and *bab*-GAL4s were analyzed at both adult and pupal stages, whereas *ap*-GAL4 was analyzed only at mid-pupal stages due to the lack of adult expression.

**Table 1:** Summary of GAL4 reporter expression of PD network TFs in ORNs.

Sensilla	ORs	Glomeruli	Expression of Prepatterning Factors				
			Ap	Bar	Bab	Rn	Dac
**at1**	Or67d	DA1			+/-	+	
**at2**	Or23a	DA3	+		+		
	Or83c	DC3			+		
**at3**	Or2a	DA4m			+	+	
	Or19a/b	DC1			+	+	
	Or43a	DA4I			+	+	
**at4**	Or47b	VA1v			+		
	Or88a	VA1d	+	+	+		
	Or65a/b/c	DL3	+				
**ab1**	Gr21a	V					
	Or92a	VA2					+
	Or10a/Gr10a	DL1			+		+
	Or42b	DM1					+
**ab2**	Or59b	DM4	+				
	Or85a	DM5					
**ab3**	Or22a/b	DM2					+
	Or85b	VM5d			+		
**ab4**	Or7a	DL5	+				
	Or56a/Or33a	DA2			+		
**ab5**	Or47a	DM3				+	
	Or82a	VA6			+	+	
**ab6**	Or49b	VA5			+		
**ab7**	Or67c	VC4			+/-	+	+
	Or98a	VM5v			+/-	+	
**ab8**	Or9a	VM3					
	Or43b	VM2	+				
**ab9**	Or67b	VA3			+		+
	Or69aA/B	D			+		+
**ab10**	Or49a/Or85f	DL4				+	
	Or67a	DM6				+	+
**ac1**	IR31a	VL2p	+		+	+	
	IR92a/IR76b	VM1			+	+	
	IR75d	VL1		+	+	+	+
	?	VM6			+	+	
**ac2**	IR75a	DP1l			+		
	IR41a/IR76b	VC3m/VC5?			+		
	IR75d	VL1		+	+		+
**ac3**	IR75a/b/c	DL2d/DL2v?			+		
	Or35a/IR76b	VC3I					
**ac4**	IR84a	VL2a		+	+	+	
	IR76a/IR76b	VM4				+	
	IR75d	VL1		+	+	+	+
**ai1**	Or13a	DC2				+	+
	Or46aB?	VA7m			+	+	+

Summary of the expression analyses shown in Fig 4 and S4 Fig. Pupal brains (50–75 hr APF) were used to examine *ap*-positive ORN identities by *ap*^*md544*^-driven GFP. Pupal and adult brains were used for analyzing the *bab* (by *bab1*^*Pgal4-2*^-driven GFP) and *Bar* (by *NP4099*-driven GFP) data. Expression data for *rn* (in pupal stage and by lineage tracing) and *dac* (in adult stage) were taken from Li *et al*. and Song *et al* [35, 38]. Weak expression is indicated by “+/-”.

#### Ring 1

**Fig 5:**
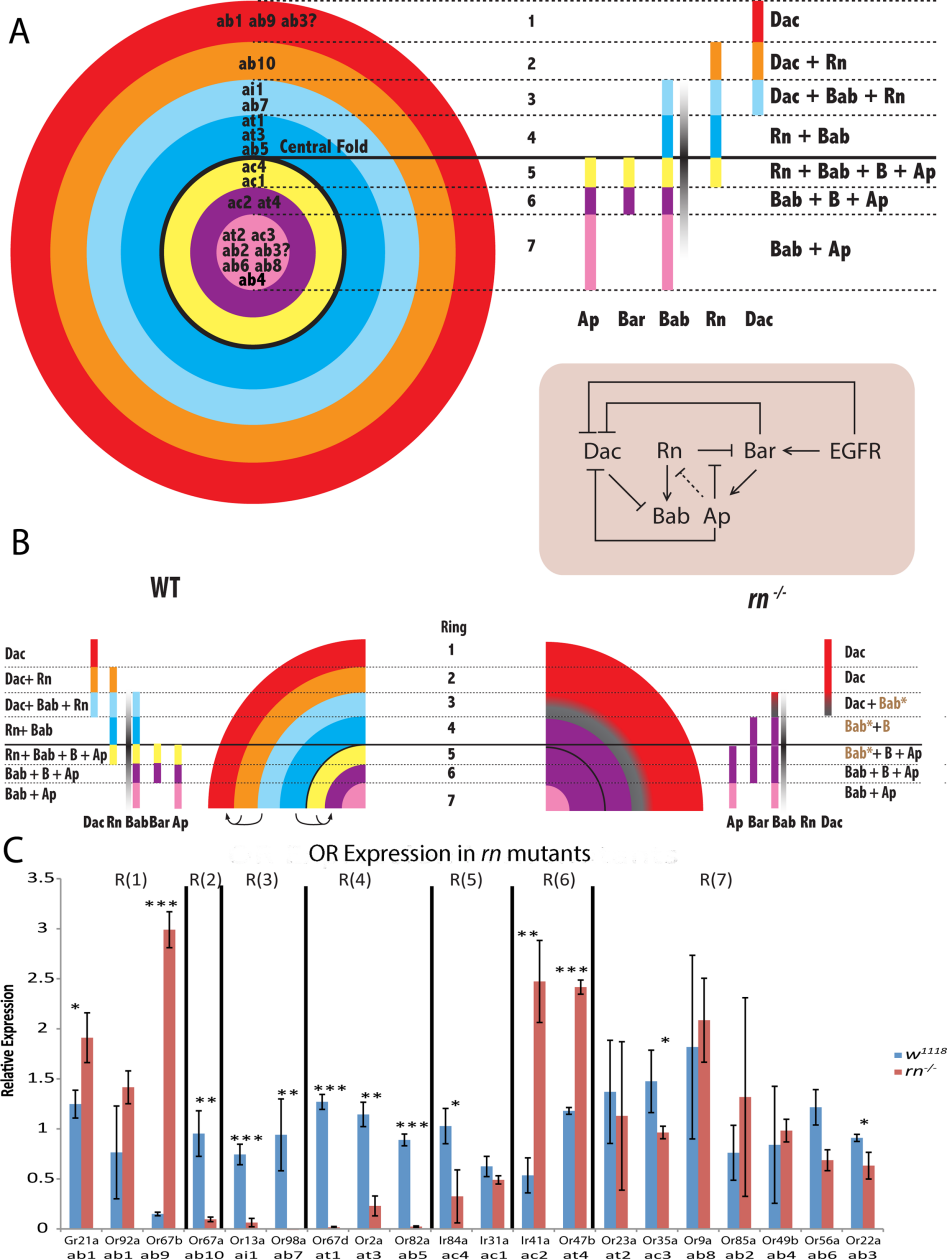
Model depicting the PD network that determines the precursor identities of the rings and the ORN populations. (A) Wild type antennal disc showing the 7 rings corresponding to subsets of sensilla subtype fates created by the combinatorial expression of the PD network components, as well as the Bab gradient. The cross-regulatory network that operates within the antennal disc is shown below. The origin of ab3 (with a question mark) is unclear. (B) Scheme showing the changes to the overlapping domains within the antennal disc in *rn* mutants. R(6) and R(1) are expanded at the expense of R(2–5). See S1 Text for explanations. (C) qRT-PCR of OR genes as a readout of ORN populations in antennal samples from *rn* mutant flies. *p<0.05, **p<0.01, ***p<0.001.

ab1, ab9, and ab3 are labeled by the *dac* reporter, but negative for *rn*, *Bar* and *ap* (Table 1), and it has been shown that Dac is required for the specification of these three sensilla subtypes [35]. Therefore, we map ab1, ab9, and ab3 to R(1) (Fig 5A). However, they are also positive for *bab* based on reporter expression in ORNs (Table 1 and Fig 4). Because there is no ring that is positive for Bab and Dac expression but negative for Rn in the 3^rd^ instar larval antennal disc based on antibody stainings (Fig 3A), we attribute this discrepancy to either an artifact of the *bab* reporter or late expression of Bab unrelated to precursor fate determination.

#### Rings 2, 3, 4, and 5

Next we wanted to map sensilla to the middle 4 rings, all of which are *rn*-positive. Because the only Rn-positive, Bab-negative and Dac-positive domain is R(2) (Fig 3A), we assigned this ring with the ab10 fate, which is the only sensilla subtype that meets the same criteria (Table 1 and Figs 4 and 5A). Likewise, ab7 and ai1 are the only two sensilla subtypes that are triple labeled by *rn*, *bab* and *dac* reporters, and are therefore mapped to R(3) (Table 1 and Fig 5A). at1, at3, ab5 are labeled by *rn* and *bab* but not by any other reporters, we assigned them to R(4) (Table 1 and Fig 5A). Finally, ac1 and ac4 are mapped to the quadruple positive R(5). Consistent with this mapping, these two sensilla are positive for *rn* and *bab*, and ac1 is labeled by the *ap* reporter, ac4 is labeled by the *Bar* reporter. Even though it seems neither of these two sensilla are labeled by all 4 factors simultaneously, the expression of these factors may be only required for precursor fate specification in a narrow window of the larval stage, and later restricted to specific lineages and daughter cells for downstream functions. Consistent with this explanation, *ap* expression is highly dynamic, and disappears in the adult stage. We also detected *dac* and *Bar* expression in IR75d ORNs in coeloconic sensilla, which is the only ORN class that is found in more than one sensilla subtypes (ac1, ac4, and ac2). We currently cannot distinguish whether this is due to a developmentally related event unique to this ORN class or an artifact of the reporter expression.

#### Ring 6

R(6) is labeled by Bar, *ap*, and Bab in the antennal disc (Fig 3A and 3C–3E). The only remaining sensillum that is unambiguously labeled by these three factors is at4. Consistent with the notion that these sensilla arise from R(6), at4 sensilla are expanded in *rn* mutants [38] and this expansion is dependent on the expansion of *Bar* expression in the disc during development (Fig 2A and 2B). The only other sensilla subtype that is left and also expanded in *rn* mutants is ac2, which is *bab*-, *Bar*-positive, and *ap*-, *rn-*negative (Table 1). Given that ac2 behaves similarly to at4 from our genetic studies (see below) and previously published results [38], we deduced that the R(6) region generates precursors that give rise to ORNs in ac2, as well as at4 sensilla (Fig 5A). The reporter line we used for *ap* may not capture its expression in ac2 ORNs properly due to the complexity of its enhancer elements.

#### Ring 7

Finally, at2, ab2, ab4, ab6, ab8, and ac3 are *rn*-negative and unaffected in *rn* mutants [38]. This would place them either in the center of the disc or on the periphery, based on whether they express *dac* (periphery) or ap/bab (center). Because all contain at least one ORN class that is positive for *ap* and/or *bab*, but they are all negative for *dac*, we assigned them to R(7) in the center (Table 1 and Figs 4 and 5A). ab3 has been placed within R(1), because of the expression of *dac* and its requirement for the development of ab3 ORNs (Table 1 and Fig 5A). However, ab3 behaves similarly to the sensilla subtypes from the center R(7) in some genetic manipulations (see the following section). We are thus unable to fully resolve the origin of ab3 sensilla. It is possible that ab3 is determined by different sets of factors, one specified in R(7), and the other one specified in the outermost R(1).

These series of analyses provide us with a sensilla subtype fate map on the concentric domains of the larval antennal disc. Each ring is labeled by a unique set of PD transcription factors, corresponding to a specific subset of sensilla subtype fates (Fig 5A). We used this model to explain the majority of the phenotypes observed in *rn* mutants and other perturbations of the network components (Fig 5B and S1 Text).

### Functional involvement of PD genes in generating ORN diversity

Our model makes predictions as to how manipulations of the patterning network would lead to changes in ORN diversity. As previously reported, *rn* mutation effectively halves the amount of ORN diversity in the antenna [38]. We constructed a scheme to depict the spatial relationships of the PD transcription factors in *rn* mutants (Fig 5B). In this model, the TF combinations in Rn-positive domains, namely R(2) to R(5), are altered due to the changes to the expression of Bar, Bab and Rn. As a result, R(1) would be expanded into R(2) and the proximal portion of R(3). Similarly, R(6) would be expanded into R(4) and R(5) in *rn* mutants (Fig 5B and S1 Text).

Because we observed an expansion of Bar in *rn* mutants and this expansion is required for their ORN phenotypes, we wanted to test the effects of ectopic Bar expression in the *rn* expression domain on ORN populations. Ap was previously shown to protect Bar from being repressed by Rn during leg development [51]. Therefore, either overexpressing Bar directly or indirectly by overexpressing Ap should at least partially recapitulate the adult ORN phenotypes in *rn* mutants. We analyzed OR expression as readouts of ORN classes using quantitative RT-PCR (qRT-PCR) for a panel of 20 olfactory receptor genes representing each of the antennal sensilla subtypes in these genetic backgrounds. We confirmed that this assay provides a reliable readout of ORN fates, by showing that the predicted OR expression profiles in *rn* mutants were readily reproduced (Fig 5C) [38].

**Fig 6:**
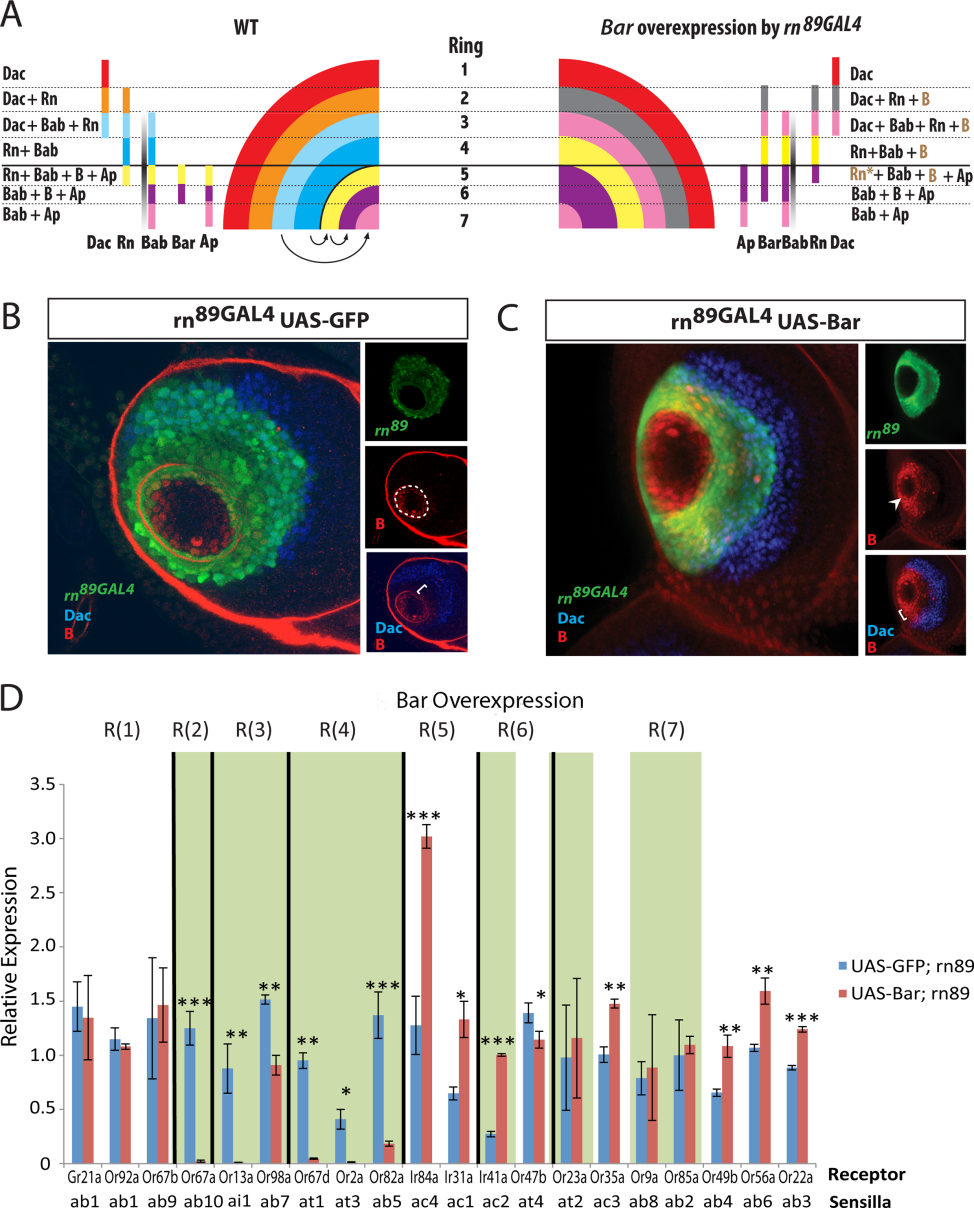
Effect of Bar overexpression on ORN populations. (A) Model depicting the changes to the combinatorial code in *rn*^*89GAL4*^ UAS-BarH1 flies. The fate conversions among the rings are represented by arrows. See S1 Text for explanations. (B) *rn*^*89GAL4*^ UAS-GFP (green), Bar (red), and Dachshund (blue) staining on antennal discs from control flies. (C) *rn*^*89GAL4*^ UAS-BarH1^M13^ flies shows expansion of Bar up to the Dac expression boundary (bracket). Some Bar is detected inside of the Dac expressing region, but this expression is weak and only present in a small number of cells. Central fold is missing in the overexpressing line (arrowhead). (D) qRT-PCR of OR genes as a readout of ORN populations in antennal samples from *rn*^*89GAL4*^ UAS-BarH1^M13^ flies. The receptors that show the same phenotype as in *rn* mutants are blocked in green shade. *p<0.05, **p<0.01, ***p<0.001.

**Fig 7:**
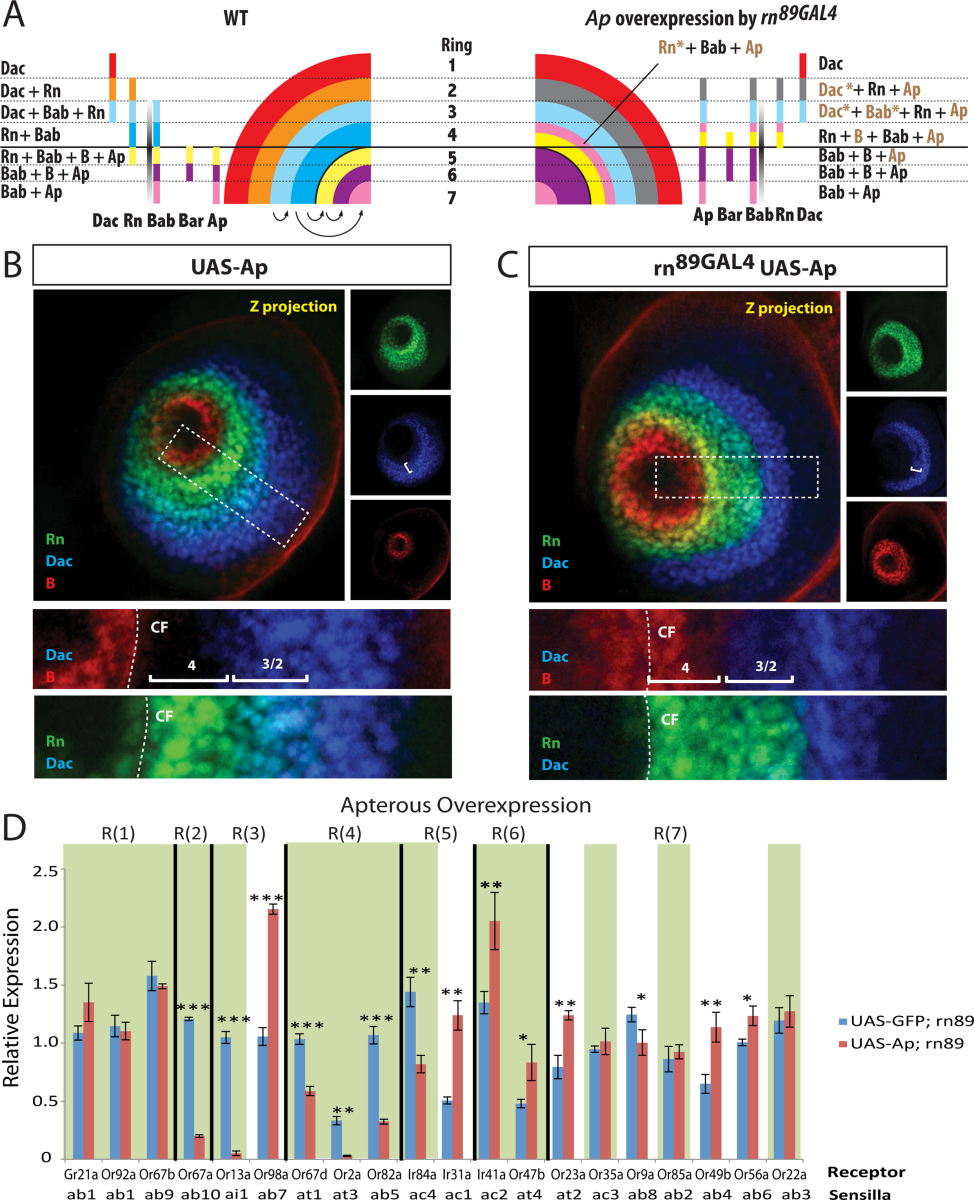
Effect of Apterous overexpression on ORN populations. (A) Model depicting the changes to the combinatorial code in *rn*^*89GAL4*^ UAS-ap flies. (B) and (C) Rn-EGFP (green), Bar (red), and Dachshund (blue) staining on antennal discs from wild type (B) and *rn*^*89GAL4*^ UAS-ap flies (C) shows expansion of Bar to R(4) and decreased Dac staining in R(2/3) (brackets). Individual channels are shown on the right. Boxed areas are shown below at a higher magnification. (D) qRT-PCR of OR genes as a readout of ORN populations in antennal samples from *rn*^*89GAL4*^ UAS-ap flies. The receptors that show the same phenotype as in *rn* mutants are blocked in green shade. *p<0.05, **p<0.01, ***p<0.001.

As predicted, when Bar or Ap was overexpressed, the changes in the expression of the majority of ORs trended towards changes observed in *rn* mutants (Figs 6D and 7D). One exception was Or47b in at4 that was downregulated in Bar overexpression lines. We have already shown, however, that the expansion of Bar expression accounts for the increase of at4 sensilla in *rn* mutants. To reconcile this discrepancy, we re-examined the expression of *Or47b* using a reporter line in Bar overexpressing flies. Although we observed an overall decrease in the number of Or47b neurons consistent with the qPCR result, the domain of expression was expanded to the medial region similar to the manner observed in *rn* mutants (S5 Fig). In agreement with the expansion of at4 sensilla, glomerular sizes appeared larger for all at4 ORNs (Or47b, Or88a, and Or65a) compared to wild type (S6A, S6B and S6D Fig), a similar phenomenon observed in *rn* mutants. (S6A and S6C Fig) [38]. In contrast, the target glomeruli are lost for ab5 ORNs, which show dramatic reduction based on OR expression in qRT-PCR (Figs 6D and 7D and S6A–S6D Fig). We further validated the expression of a subset of ORs in the antenna using reporter lines, we could recapitulate the same changes in OR expression uncovered by the qPCR analysis (S6E–S6M Fig). Of particular note is that ORs in the same sensillum (ab10: Or49a, ab7: Or67c) changed in a similar manner to their partner OR genes (ab10: Or67a, ab7: Or98a) (S6E–S6M Fig and Figs 6D and 7D). These results suggest that manipulating the PD gene network causes switches of SOP fates and ORN populations.

During our examination of Bar-overexpressing larval antennal discs, we found that the central fold (CF) disappeared (Fig 6B and 6C and S7 Fig). In contrast, *ap* and Bab expression patterns were unaffected (S7 Fig). Similarly, Dac showed normal expression, despite the reported function of Bar to repress Dac in the distal area, which suggests that the repression may be time-sensitive and/or context-dependent [47,52].

Next we examined the effects of Ap overexpression on the expression patterns of the network genes in the antennal disc. As expected, Ap overexpression resulted in the ectopic expression of Bar protein outside of its normal boundaries in the antennal disc (Fig 7B and 7C and S8A and S8B Fig). Similar to *rn* mutants, an expanded Bar zone is bounded by the distal limit of Dac in this background (Fig 7B and 7C). However, unlike in *rn* mutants, Bar does not fully extend to the boundary, and hence, these proximal cells in R(4) are positive for Rn but negative for Dac and Bar (S8D Fig). They also express Bab and Ap, making them a separate subpopulation within R(4). In addition, we saw a loss of Rn expression in R(5), leaving the domains within the central fold devoid of Rn expression (S8A–S8C Fig). The simplest interpretation of this data is that increased levels of Ap repress Rn expression in a context-dependent manner. Moreover, we found that Dac expression is decreased in R(2) and R(3) that also express Rn (Fig 7B and 7C), suggesting that *rn* promoter-mediated Ap expression represses Dac in this overlapping domain. Because Dac represses Bab [57], the reduction in Dac expression should theoretically cause an increase in Bab expression, although we cannot detect any obvious changes for Bab. This may be due to its overall low concentration in this region by repression from other factors [57].

Based on these analyses, we drew similar illustrations for precursor domains in the Ap and Bar overexpression backgrounds (Figs 6A and 7A and S1 Text). They reveal different patterns of gene expression for a number rings compared to the *rn* mutants, which may account for their differences in adult ORN classes as shown by the qPCR results (Figs 6D and 7D). We conclude that the PD gene regulatory network function in combinations to diversify precursor and ORN fates.

We next examined the requirement of Bar in producing the four fates that arise from the Bar-positive region (Fig 5A). To do this, we created *Bar* mutant clones that delete both *BarH1* and *BarH2*. However, our analysis did not reveal any significant changes in adult OR expression (S9A Fig and Fig 2C and 2D). The most likely explanation for this observation is that Ap and Bar may have partially redundant functions for some sensilla subtypes, such as at4 and ac2 in R(6). Consistent with this, transheterozygous *ap* mutant alleles using *ap*^*md544GAL4*^ and the *nap1* deficiency did not affect ORNs from at4 and ac2 precursors, either (S9B Fig). In fact, only three sensilla subtypes (ab2, ab6, and ac1) from R(7) and R(5) showed modest decreases in OR expression in *ap* mutants (Fig 5A and S9B Fig). These results suggest that the developmental refinement of SOP fates in the three inner rings are robust, which makes their dependence on factors like Bar and Ap limited.

It has been shown in the leg that this network of PD genes functions under the control of an EGF signaling gradient, which is highest at the center of the disc and decreases outward [33]. There, EGFR signaling represses Rn and activates Bar expression [33,49]. We next tested the hypothesis whether perturbations in EGFR signaling can cause modifications to ORN fates. To do this, we expressed a constitutively active EGFR [33] using *rn*^*89GAL4*^ and performed qRT-PCR on ORs (S10A Fig). As expected, these experiments showed that ectopic activation of EGFR function is associated with an expansion of Bar and reduction of Rn expression in the antennal disc (S10B and S10C Fig). In addition, the ORN classes originated from R(1), R(2), R(3), and R(5) precursor domains were affected in the adult. These results suggest that EGFR signaling may indirectly regulate ORN diversity by modulating the PD gene network.

### Separation of different precursor fates within a ring by Bab concentration gradients

**Fig 8:**
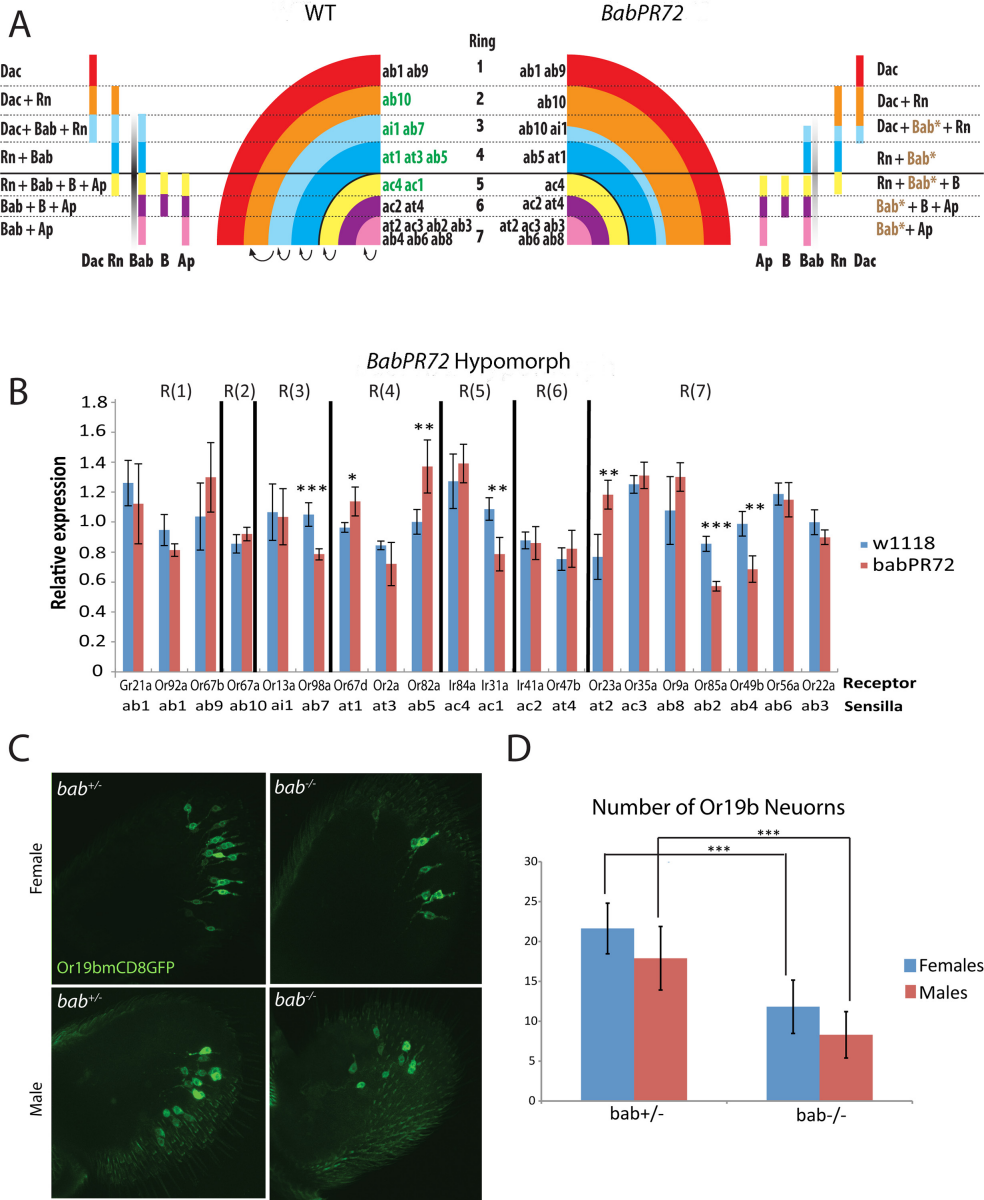
Effects of hypomorphic *bab* mutants on ORN populations. (A) Model depicting the changes to the combinatorial code in *bab*^*PR72*^ homozygous flies. The fate conversions among the rings and ORN pools are represented by arrows. (B) qRT-PCR of OR genes as a readout of ORN populations in antennal samples from *bab*^*PR72*^ homozygous flies. The 8 *rn*-positive ORs are from R(2)-R(5) rings. * *p*<0.05, ** *p*<0.01, *** *p*<0.001. (C) Antennal images of Or19b (at2) expression in control (left) and *bab*^*PR72*^ mutant (right) flies. The top panel is females and the bottom panel is males. (D) Quantification of the number of Or19b neurons from (C). Both males and females show a significant reduction in the number of neurons. *** *p* < 0.001.

Bab is partially activated by Rn, and it is significantly downregulated in *rn* mutants (Fig 1D and 1E). It is plausible to think that Bab functions downstream of Rn to specify *rn*-positive precursor fates. If this is the case, we should see reduced expression of the receptors from the eight *rn*-positive sensilla subtypes in a *bab* mutant. To our surprise, only two of the eight receptors tested showed reductions, and another two were even increased in the *bab*^*PR72*^ hypomorphic allele (Fig 8B).

We noticed a range of changes for sensilla subtypes from the same ring (Fig 8B). For example, among sensilla specified in R(7), ab2 and ab6 are reduced, whereas at2 is increased in the *bab* mutant. The simplest interpretation is that different levels of Bab are required to distinguish these fates in the same ring. When the overall level of Bab is decreased, some sensilla requiring higher Bab may be converted to the ones that require lower Bab. Similarly, only ac1 from R(5) is reduced, and one explanation is that the lowered Bab expression is still above the threshold for specifying ac4, but not for ac1. Alternatively, ac1 (requiring higher Bab) may be converted to ac4 (requiring lower Bab), and compensates for the loss of the endogenous ac4, which may die due to the reduction of Bab. The same reasoning can be applied to ai1 versus ab7 from R(3). For R(4), we saw increases in at1 and ab5, and a trend towards downregulation for at3 in the mutant, albeit the latter was not significant (Fig 8B). We then counted the number of Or19b neurons housed in at3 sensilla, and found that it is significantly reduced (Fig 8C and 8D). This discrepancy in the qPCR result may be due to the random fluctuation of gene expression levels, especially when the changes in the numbers of cells are small. This result suggests that similar conversions may occur in R(4) among the three fates when Bab is reduced. In contrast, the Bab-positive at4 and ac2 from R(6) appear to be normal in this hypomorphic allele. This could either be because these two sensilla are specified with wider ranges of Bab levels or some other factors are needed to differentiate the two fates. Taken together, these data suggest that Bab could be an essential factor to distinguish alternate SOP fates within a ring using its concentration gradient.

### Functionally conserved molecular network in patterning gustatory receptor neuron fates

The distal portions of both the legs and antennae are chemosensory organs covered by sensilla. The legs, being part of the gustatory system, display neuronal and molecular diversity that is characterized by a huge variety of gustatory receptors expressed on the legs. Unlike ORNs, individual gustatory receptor neurons (GRNs) express multiple receptors, and a given GRN class can be found in different sensilla within different GRN clusters [32].

Because the legs and antennae use the same molecular network to pattern these chemosensory appendages, we asked if a similar genetic program operates to pattern the adult GRN fates. We tested this hypothesis in *rn* mutants as *rn* is thought to be required for the development of tarsal segment 3 (ta3), and this segment is lost in *rn* mutants [58]. However, we do not have a reporter line that uniquely labels ta3.

**Fig 9:**
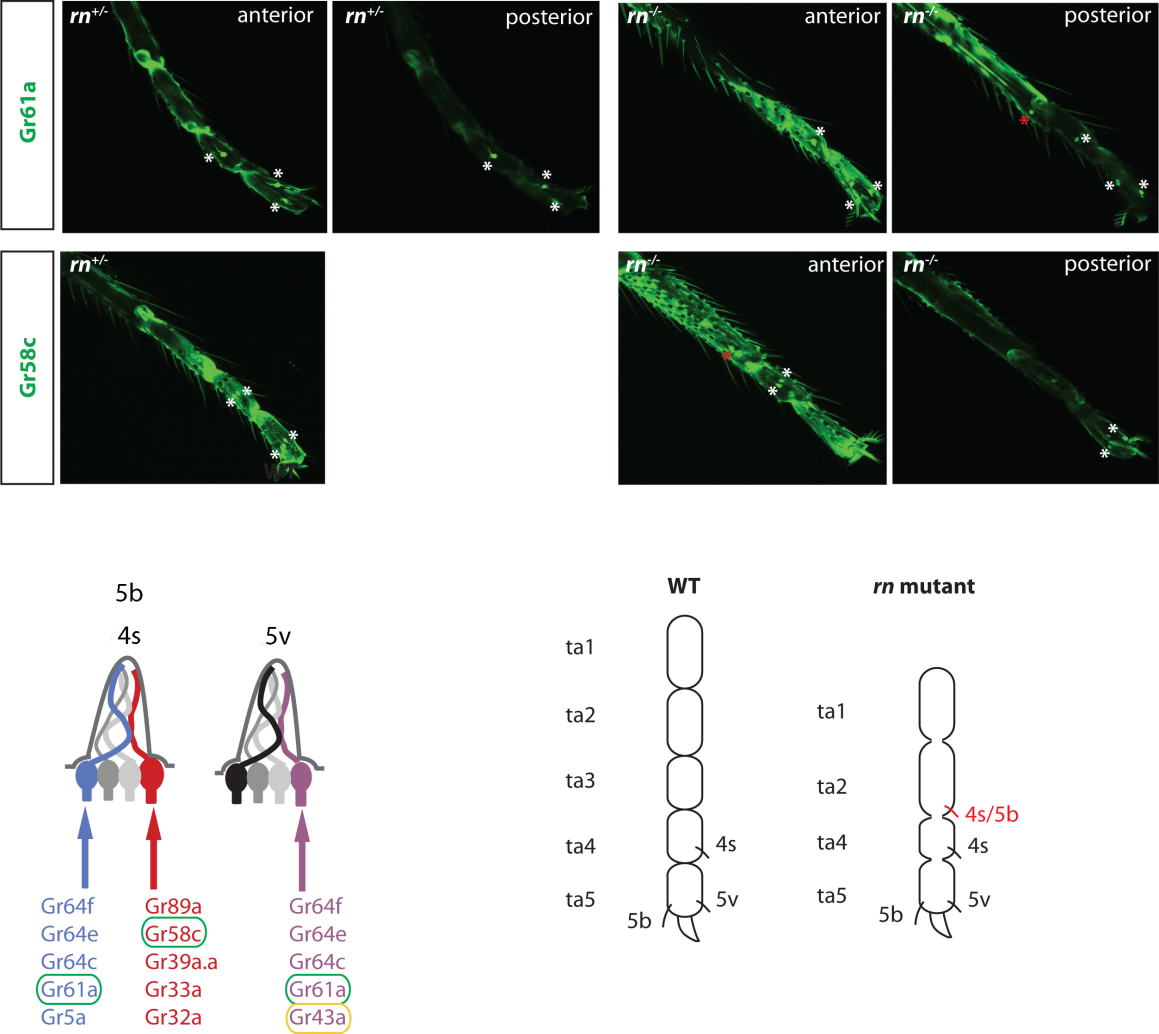
Regulation of GRN patterns by the PD network. GRN and ORN specification may be regulated by the same molecular network. In *rn* mutant, tarsal segment (ta3) is missing, and other segments are fused. Ectopic expression of Gr61a and Gr58c can be seen in the distal region of the second tarsal segment, suggesting a duplication of 4s/5b sensilla. Neurons labeled by the red asterisk in the pictures are the ectopic neuron only seen in the mutant. Different sections of confocal images are shown when neurons are not on the same focal plane. All legs are either from the mid or from the hind leg. Cartoon illustrating the *rn* phenotype in GRNs is shown below. The ectopic sensillum is labeled in red. The scheme for GRN classes and the receptors expressed in the 5b/4s and 5v sensilla are modified from [32]. The GR that distinguishes 5b from 5v is circled in yellow (also see S9 Fig). Receptors that are analyzed in the figure are circled in green. In the wild type, there is a pair of sensilla for each sensilla type, although only one for each pair is drawn in the cartoon.

On the other hand, Gr5a and Gr61a are expressed in the mid and hind legs, where they are restricted to the GRNs in ta4 and ta5. In both cases, we could reproducibly detect an extra neuron in the mid or hind legs of *rn* mutant (Fig 9 and S11 Fig). To confirm this result, we used a reporter to label the bitter receptor Gr58c that is expressed by a partner neuron in the same sensilla. We observed ectopic Gr58c neurons in *rn* mutants (Fig 9). In contrast, the Gr43a-expressing neurons, which coexpress Gr61a but are housed in another sensillum appeared be unchanged in the mutant (S11 Fig). These results suggest that the sensilla, 5b and 4s, that house the Gr5a/Gr61-expressing sugar neurons and the Gr58c-expressing bitter neurons are expanded towards the proximal segment of the legs in *rn* mutants (Fig 9). Taken together, we speculate that the same molecular network is used in parallel to diversify chemosensory neurons in the antennae and legs.

## Discussion

How neuronal diversity in the brain is generated from a limited genomic toolkit remains largely unknown. In the *Drosophila* olfactory system, selective expression of typically a single olfactory receptor gene from a repertoire of approximately 80 possible genes generates 50 different classes of ORNs. ORN classes are found in invariable clusters of 1–4 neurons in individual sensilla, which can be classified into types based on their morphology and subtypes based on the specific combination of ORN classes they house [7,12,20]. Here, we show that a functionally conserved cross-regulatory transcription factor (TF) network module patterns the ORN precursor field along the proximodistal axis prior to neurogenesis. The interactions between different components of the TF network module partition the precursor field into concentric domains in response to an EGF signaling gradient. These domains represent clusters of epithelial cells with distinct differentiation potentials, which are defined by unique combinations of TFs that ultimately drive specialization of these cells into sensilla subtype lineage-specific SOPs. Genetic manipulations of the network alter this combinatorial code and lead to predictable shifts between sensilla subtypes and neuronal identities. Our results suggest that this early TF network plays a major role during neuronal diversification by prepatterning the antennal disc, thereby restricting the identities of cells in the precursor field.

Although our model of sensilla fate mapping can explain the majority of sensilla subtype specification and consequent ORN diversity, it is likely incomplete. For example, some distinct sensilla subtypes within a ring are specified by the same set of factors suggesting that additional/unknown genes must be contributing to the differentiation process. One possibility is that factors that establish the dorsal/ventral (D/V) axis or anterior/posterior axis, such as *engrailed* and other prepatterning genes such as *lozenge*, can be superimposed onto the presented network and used in this process. These factors might function to regulate the expression of proneural genes, *atonal* and *amos*, adding an additional spatio-temporal aspect to regulate the selection of SOPs from prepatterned fields, as in the case of *amos* being regulated by *lozenge* [40,44,59,60]. Future work should address the contribution of these axis determination events to ORN diversification. The current study mainly focused on the final phase of the prepatterning stage, and hence the functional relevance of the temporal aspect of the TF network deserves further investigation.

### A common strategy with simple logic steps for generating neuronal complexity

We propose a conserved stepwise strategy to explain the overall ORN diversity. First, the prepatterning phase generates distinct pools of epithelial cells with unique differentiation potentials. This is followed by sensory organ precursor selection by proneural genes. Finally, these precursors undergo neurogenic divisions that allocate alternate fates into daughter cells through Notch signaling and terminal selector transcription factors. One salient aspect of this cellular diversification strategy is its modularity. Each step is driven by context-independent rules, yet produces vastly different neuronal outcomes across systems in a developmentally context-dependent manner. For example, Rn is used to generate distinct precursor differentiation potentials in both the antennal and leg discs to increase the complexity of the patterned precursor fields, which give rise to ORNs and GRNs, respectively. Similarly, Notch is reiteratively used during SOP divisions to generate each sensillum. Its function of segregating binary cell fates is context-independent, although the exact fates being segregated are quite different for each sensillum [61]. Therefore, this stepwise mechanism simplifies the overall difficulty of creating neuronal diversity all at once by logically deconstructing similar differentiation processes into single-purpose steps with shared control elements.

Even though our findings are in the PNS, there are similar examples of stepwise patterning and diversification in the fly CNS. For example, different neuroblast lineages in the *Drosophila* embryonic CNS are first specified by spatially restricted factors within specific positions of an orthogonal grid in the embryo [62]. Anterior-posterior axis specification is controlled by Hox-segment polarity genes, which determine the overall fate, just as in the PNS (leg vs. antenna). Dorsoventral patterning is controlled by cross-regulatory transcription factors, which are turned on in response to different concentrations of morphogens such as Hedgehog and Dpp. Similar to olfactory SOPs, patterning of the neuroepithelium is followed by the expression of proneural genes and selection of neuroblasts, which undergo asymmetric divisions and neurogenesis. The division patterns and factors that are asymmetrically segregated into each daughter cell are remarkably similar regardless of the neuroblast lineages to which they belong [62,63].

There are also parallels between our findings in the fly olfactory system and the more complex vertebrate olfactory system. Even though stochastic selection has been proposed as a mechanism for the expression of specific OR genes by different ORNs, the restriction of mammalian OR expression into distinct zones suggests that a deterministic mechanism may also be at play [21]. Consistent with this hypothesis, some OR classes that are restricted to specific domains in the mammalian olfactory epithelium were shown to contain known TF binding sites [64,65]. Interestingly, some TFs, such as the mammalian orthologue of Apterous, Lhx2, have evolutionarily conserved developmental functions in olfactory neurons [66]. We suspect that some of the mechanisms used in diversifying fly ORNs may also be used in the mammalian system during the step of OR zonal separation.

Examples of similar neuronal diversification cascades utilizing gene regulatory networks under morhogen gradient control are also seen in the vertebrate CNS and PNS. In the classic example of spinal cord neuron diversification, morphogen gradients (BMP/Shh) along the D/V axis of the neural tube activate different sets of transcription factors in the precursors to set up a number of domains prior to neurogenesis, thereby diversifying both progenitor and sensory neuronal subtypes they generate [67,68]. Recently, the radial glia that give rise to neurons of the cortex were also found to be heterogeneous [69]. Such combinatorial TF network modules confer positional and temporal information to each neural stem cell in order to create a diverse progenitor population in the mammalian cerebral cortex [69]. Segmental patterning of these neural stem cells contributes to neuronal and glial diversity [70]. Similarly, cortical projection neuron fates can be switched among lineages when the corresponding gene network is modified, changing the zonal partitioning of the neocortex [71]. These results, in light of our findings, point to a common strategy composed of modular and simple commands functioning in a nested manner to increase neuronal diversity in multiple developmental contexts.

### ORN precursor fate specification and OR expression

We associate terminal differentiation of ORNs with the specific selection of an OR gene for expression. At least in flies, it is possible that OR expression and ORN diversity are regulated by a set of “terminal selector genes,” similar to those proposed by Oliver Hobert [72]. Here, a TF, or combination of TFs, directly regulates the expression of genes required for terminal differentiation and function [72]. So far, a list of postmitotic TFs have been shown to directly regulate OR expression by associating with OR promoters [73–77]. However, the loss of these TFs only affects the expression of specific subsets of OR genes, and yet most OR genes have binding sites for these factors. The functional specificity of each TF and their expression patterns in ORN classes have not been well defined. It is possible that chromatin states around OR promoters in different ORN classes govern how these TFs function within each class. Epigenetic modulations of chromatin status have been shown to play an important role in numerous developmental processes, including the development of the olfactory system [78–82]. The prepatterning TFs could recruit epigenetic modifying factors to change the open and closed states of the chromatin around genes critical for different fates. These modifications can be inherited during cell divisions, and affect the genomic accessibility of later factors. Regardless of the exact mechanisms in between, it is likely that the expression/function of terminal selector genes are regulated, at least in part, by the developmental context established by the prepatterning network that we have described. Establishing a clear link between the early patterning networks and the late terminal selector TF network will be critical to resolving these paradoxical results.

### Deployment of the same network module in diversifying olfactory and gustatory neurons

This patterning network is remarkable in its functional and structural similarity in driving neuronal diversity in two related chemosensory organs, the antennae (olfactory) and the legs (gustatory). At the top of the hierarchy of the cascade, Hox genes determine the overall neuronal identities within the discs during embryogenesis. Olfactory lineages, for instance, are determined by the gene *homothorax* in the antennal disc, whereas *homothorax* is inhibited by Antennapedia in the leg discs conferring gustatory identities [83]. Strikingly, regardless of the particular Hox gene, the PD gene network module seems to perform similar diversification commands in both chemosensory systems. It will be interesting to ask how different sets of identity genes are regulated in different tissues on the molecular level, and how the cellular memory is passed down through the cascade.

### Modular evolution of gene regulatory networks and neuronal diversity

Understanding the diversification process from homogeneous fields of precursors to diverse, terminally differentiated neuronal populations will provide key insights into how cascades of master regulatory transcription factor networks can generate and modify the cellular complexity seen in multicellular organisms. Understanding this diversification process can also help us understand the origins of this complexity. At an evolutionary scale, clear analogy exists between ORN precursor diversification process and the segment diversification during early embryogenesis along the myriapods-insect lineage [84]. The addition or elimination of TFs governing either process might reflect, or likely instruct the generation of new fates. Based on a modern version of “the law of development” postulated almost two centuries ago [85], the acquisition of increased complexity of a tissue and the concomitant genetic changes over evolutionary time is recapitulated by the temporal role and developmental order of the genes that establish the complexity. Under this assumption, a primordial state in antennal development might be the expression domains of Bar and Dac in the antennal disc. Rn was then added to this network later in development and evolution. This would explain the dramatic decreases in ORN diversity and the expansion of specific ORN populations in default sensilla subtypes in *rn* mutants as well as Bar/Ap overexpression. Indeed, the onset of Rn expression is later in third instar discs compared to those of Dac/Bar/Ap [51], and Rn seems to be unique to Arthropods, especially insects. Thus, it is plausible that Rn is a newer addition to the network. Conceivably, Rn evolved to generate novel olfactory neurons in order to help the ancestral Arthropods exploit novel olfactory niches.

## Materials and Methods

### Fly genetics

bab^A128^, Df(3L)bab^PR72^, were from Frank Laski. Df(1)^B263-20^ FRT19A, UAS-BarH1^M13^ were from Tetsuya Kojima. UAS-Egfr.λtop4.4 was from Amanda Simcox. rn^tot^, rn^tod^ was previously described [38]. OR-CD8 GFP, OR-GAL4, IR-GAL4, GR-GAL4 lines were from Leslie Vosshall, Barry Dickson, Richard Benton and John Carlson, respectively [7,20]. Or67d^GAL4^ knock-in stock was a published line showing faithful expression of Or67d [86]. rn^89^, bab1^Agal4-5^ (#6802), bab1^Pgal4-2^ (#6803), ap^md544^, ap^rK568^, UAS-ap, Df(2R)nap1, tubP-GAL80 ey-FLP FRT19A, FRT19A, UAS-CD8 GFP, UAS-Syt GFP, UAS-FLP were all from Bloomington Stock Center. NP4099 (Bar^GAL4^) was from Drosophila Genetic Resource Center.

Genotypes for fly genetics:

Fig 1B. w^1118^. ap^rk568^.Rn-EGFP

Fig 1C–1E. rn^+/-^: UAS-CD8 GFP/+; rn^89GAL4^/TM6b. rn^-/-^: UAS-CD8 GFP/+; rn^89GAL4^/rn^tod^

S3A Fig ap^rK568^/UAS-CD8 GFP; rn^89GAL4^/rn^tod^

S3B Fig bab1^A128^/+

S3C Fig Rn-EGFP

Fig 2A. Bar^+/-^ rn^+/-^: eyFLP FRT19A Tub^GAL80^/+; Or47b::mCD8-GFP/+; rn^tot^/+. Bar^+/-^ rn^-/-^: eyFLP FRT19A Tub^GAL80^/+; Or47b::mCD8-GFP/+; rn^tot^/FRT rn^tot^. Bar^-/-^ rn^-/-^: eyFLP FRT19A Tub^GAL80^/ Df(1)^B263-20^ FRT19A; Or47b::mCD8-GFP/+; rn^tot^/FRT rn^tot^

Fig 2C. Control: eyFLP FRT19A Tub^GAL80^/FRT19A; Or47b GAL4 UAS-GFP/+. Bar MARCM: eyFLP FRT19A Tub^GAL80^/ Df(1)^B263-20^ FRT19A; Or47b GAL4 UAS-GFP/+

Fig 2E. w^1118^. Rn-EGFP

Fig 3B and 3C. Rn-EGFP

Fig 3D and 3E. ap^rK568^/+, Rn-EGFP

Fig 3F. ap^rK568^/+

Fig 4A–4C and S4A and S4B Fig. NP4099 (Bar^GAL4^); UAS-CD8GFP. ap^md544^; UAS-CD8 GFP. bab1^Pgal4-2^/UAS-CD8 GFP. NP4099 (Bar^GAL4^); UAS-Syt GFP. bab1^Pgal4-2^/UAS-Syt GFP.

Fig 5C. w^1118^. rn^tot^/FRT rn^tot^

Fig 6B. UAS-CD8GFP/+; rn^89GAL4^/TM6b

Fig 6C. UAS-CD8GFP/+; rn^89GAL4^/UAS-BarH1^M13^

Fig 6D. UAS-CD8GFP/+; rn^89GAL4^/TM6b. UAS-CD8GFP/+; rn^89GAL4^/UAS-BarH1^M13^

S5 Fig Or47b::mCD8GFP/+; rn^89GAL4^/TM6b: Or47b::mCD8GFP/+; rn^89GAL4^/UAS-BarH1^M13^

S6C Fig rn^89GAL4^/rn^tod^.

S6E–S6M Fig OR::mCD8GFP. OR::mCD8GFP; rn^89GAL4^/UAS-BarH1^M13^. OR::mCD8GFP; rn^89GAL4^/UAS-ap

S7A Fig Rn-EGFP UAS-BarH1^M13^/TM6B

S7B Fig Rn-EGFP UAS-BarH1^M13^/rn^89GAL4^

S7C Fig UAS-BarH1^M13^/TM6B

S7D Fig UAS-BarH1^M13^/rn^89GAL4^

Fig 7B and S8A and S8C Fig. UAS-ap/+; Rn-EGFP/TM6b

Fig 7C and S8B–S8D Fig UAS-ap/+; Rn-EGFP/rn^89GAL4^

Fig 7D. UAS-CD8GFP/+; rn^89GAL4^/TM6b. UAS-CD8GFP/+; rn^89GAL4^/UAS-ap

S9A Fig eyFLP FRT19A Tub^GAL80^/FM6. eyFLP FRT19A Tub^GAL80^/ Df(1)^B263-20^ FRT19A

S9B Fig w^1118^. Df(2R)nap1/ ap^md544^

Fig 8B. w^1118^: bab^PR72^

Fig 8C. Or19b::mCD8GFP/+; bab^PR72^/TM6b: Or19b::mCD8GFP/+; bab^PR72^

S10 Fig UAS-CD8GFP/+; rn^89GAL4^/TM6b. UAS-CD8GFP/+; rn^89GAL4^/UAS-Egfr.λtop4.4

Fig 9 and S11 Fig rn^+/-^: GR-GAL4/UAS-CD8 GFP; rn^tot^/TM6b. rn^-/-^: GR-GAL4/UAS-CD8 GFP; rn^tot^/FRT rn^tot^.

### RNA extraction and library preparation

For the RNAseq analysis, wandering third instar larval antennal discs (~70 for each genotype), 8hr APF pupal antennae (~50 for each genotype), 40hr APF pupal antennae (~50 for each genotype), and adult antennae (150 males and 150 females) from w^1118^, rn^tot^/TM6b, and rn^tot^/rn^tot^ flies were dissected. RNA was extracted with RNeasy kit (Qiagen) following manufacturer's instructions, and was treated with on-column DNase digestion (Qiagen). We extracted RNA only from the antennal portion of the larval eye-antennal discs in order to remove contamination by transcripts from the developing eye. All samples were diluted to 20ng/ul in 55ul volume with H_2_O, out of which 3.5ul was used for quality control using Bioanalyzer (Duke Microarray Core Facility). The concentrations were measured again with Qubit 2.0 (Life Technologies), and 700ng RNA was diluted to 50ul total volume with H_2_O for each sample. RNA sequencing libraries were prepared with TruSeq Stranded mRNA Sample Prep Kit (Illumina) following the manufacturer's instructions. For the RNA fragmentation step, 94°C, 2min was used with the intention to obtain a median size ~185bp. PCR amplification was done with 15 cycles. A total of 24 multiplexed libraries (barcoded) were accessed for quality and mixed altogether before separating to two identical pooled libraries, which are subject to cluster generation followed by Illumina 50bp paired-end sequencing by UNC High-Throughput Sequencing Facility (HTSF).

### RNAseq analysis

*Drosophila melanogaster* transcriptome (r5.57) was downloaded from flybase and bwa indexed was created with bwa-0.7.8. Each sequencing file was aligned to the transcriptome, and.sam files for each sample were generated by putting two alignments from both reads together. At least over 80% of the total reads were able to align to the reference. After that, count tables were made for each sample with a customized python script, and further consolidated into a matrix containing transcript ID and read counts from all genotypes for each stage with a Ruby script. These matrices were used as inputs for differential expression analysis using a customized DESeq2 R script.

For each stage, we first filtered out ORs/IRs/GRs from the RNAseq datasets, and excluded the genes with low expression levels in all three genotypes (normalized count < 20). We then narrowed the analysis down to genes that show the same trend of differential expression when comparing homozygous vs. *w*^*1118*^ and homozygous vs. heterozygous datasets. Because the heterozygous background may have some dominant effects due to the presence of the balancer chromosome and the heterozygous flies do not show any OR phenotypes [38], saving genes that pass both comparisons would help remove irrelevant genes modified by the balancer chromosome and meanwhile enhance the discovery confidence. Because of these stringent filtering steps, we could maximize our gene lists with a more relaxed cutoff (unadjusted *p* < 0.1) for the gene ontology (GO) and functional clustering analyses.

### Immunohistochemistry

Samples were fixed with 4% paraformaldehyde, washed with phosphate buffer with 0.2% Triton X-100, and staining as previously described [87]. Primary antibodies were used in the following dilutions: rabbit α-GFP 1:1000 (Invitrogen), chicken α-GFP 1:700 (Aves Labs), rat α-Ncad 1:20 (Developmental Studies Hybridoma Bank), mouse α-Bruchpilot 1:20 (Developmental Studies Hybridoma Bank), mouse α-CD2 1:1000 (Serotec), mouse α-Dac 2–3 1:20 (Developmental Studies Hybridoma Bank), rabbit α-β galactosidase 1:800 (Invitrogen), mouse α-β galactosidase 1:800 (Promega), rat α-Bab2 1:1500 (Frank Laski), rabbit α-Bar-H1 1:100 (Tetsuya Kojima). The following secondary antibodies were used: Alexa 488 goat α-rabbit 1:1000, Alexa 488 goat α-chicken 1:1000, goat α-mouse-Cy3 1:100, goat α-rat-Cy3 1:200, goat α-rabbit-Cy3 1:200, Alexa 568 goat α-mouse IgG highly cross-adsorbed 1:300, Alexa 647 goat α-rat 1:200, Alexa 633 goat α-mouse 1:200, Alexa 647 goat α-mouse 1:200. Confocal images were taken by an Olympus Fluoview FV1000 or Zeiss LSM 510 (Light Microscopy Core Facility).

### Real-time RT-PCR

Antennae from approximately 50 flies were dissected for each genotype and at least three biological replicates were analyzed for each genotype. RNA was extracted with an RNeasy kit (Qiagen), treated with on-column DNase digestion (Qiagen), and then reverse transcribed into cDNA using the SuperScript First-Strand Synthesis System for RT-PCR (Invitrogen). qPCR was performed using the FastStart Universal SYBR Green Master Mix (Roche) or the FastStart Essential DNA Green Master Mix using standard protocol. Expression for each gene was analyzed in triplicate. Ct values were used to calculate dilution factors for each gene based upon standard curves created for each gene. Dilution factors were then normalized to the average factor of all ORs tested. See Table 2 for Primers used.

**Table 2:** qPCR primers for olfactory receptors.

Primer Name	Sequence	Amplification Efficiency
Or67d-qPCR-F	GCATCAGCTGTATACTAGAATGCTT	1.004581
Or67d-qPCR-R	GGGCCAGGCTTTCATAAAGAT	
Or23a-qPCR-F	ACTGTACCTGATCTCCGAGC	1.022281
Or23a-qPCR-R	GTCACATCGAGTAATCTATACAGCG	
Or2a-qPCR-F	CCTTCTACGATTGCAACTGGAT	1.05291
Or2a-qPCR-R	AACCTCATGACCTGCTCGAAG	
Or47b-qPCR-F	CAAATCTCAGCCTTCTGCGG	1.012215
Or47b-qPCR-R	GATACTGGCACAGCAAACTCA	
Gr21a-qPCR-F	CCAACATGTACGGCATGTACT	0.9796537
Gr21a-qPCR-R	ACAGACCCACCTCCTTGTAG	
Or92a-qPCR-F	TGATATCTTCAAGCTCTCGGATTG	0.9533596
Or92a-qPCR-R	TAGGCGGTCTTATAGAGGCG	
Or85a-qPCR-F	GAGCGACGATACAGAACCAC	0.9776528
Or85a-qPCR-R	AAGCGAACTTGGCCATCTTTAT	
Or22a-qPCR-F	CCGATCGTCGCTACAAATCC	1.034769
Or22a-qPCR-R	ATGCCAGCTTCACCATAGCC	
Or56a-qPCR-F	TTGACAGTTGGCGTTCCAAG	0.9354389
Or56a-qPCR-R	AAGCAAGGCTCAGTTCATCG	
Or82a-qPCR-F	CTCCAATTGGCATCTGGCTT	0.943322
Or82a-qPCR-R	GTTCGACAGATCCCAACGAAA	
Or49b-qPCR-F	ACAAGGTGGGAAAGTTGATGG	1.008808
Or49b-qPCR-R	AATGGCAGGACTCTTTCGCT	
Or98a-qPCR-F	ATTCAAGCCGCAGTTACAAGT	0.956284
Or98a-qPCR-R	TGCCAGCTTAGCCACCTTAAT	
Or9a-qPCR-F	GAGACCAACTGGACCGACTT	0.9326372
Or9a-qPCR-R	GAACAATCGTCGAGAAGGTGG	
Or67b-qPCR-F	AGATCGTTTGCATGCCTGTT	1.068821
Or67b-qPCR-R	GCCGGGTAAGTCAAGGTCAT	
Or67a-qPCR-F	ATTCAAGCCCATAACGCACC	1.045276
Or67a-qPCR-R	TCATCAGATCAGTGAGTCGAAGT	
IR31a-qPCR-F	CGAGATCTGTGATCTGCGTG	0.9775197
IR31a-qPCR-R	CCTGGGCATTACACATAGCTG	
IR41a-qPCR-F	CCAAATTGATTCATCTGCCGC	1.020108
IR41a-qPCR-R	ACCACGAGTACATAGCTCCAA	
Or35a-qPCR-F	CGACTTGGCCTTTACTACGGA	0.9652927
Or35a-qPCR-R	AGGGCTTGCTGTTCATCTCA	
IR84a-qPCR-F	CAGTTGGTCAGGTGTGATGG	0.9322729
IR84a-qPCR-R	AAAGTGGATGTTCTGGGTGTG	
Or13a-qPCR-F	CAATCGTTCACGCCAACAAC	0.9915544
Or13a-qPCR-R	ATCGAGGTACTTAGAATGGCCG	
ACT5C-qPCR-F	GGCGCAGAGCAAGCGTGGTA	1.029591
ACT5C-qPCR-R	GGGTGCCACACGCAGCTCAT	
GAPDH2-qPCR-F	CGTTCATGCCACCACCGCTA	1.053987
GAPDH2-qPCR-R	CCACGTCCATCACGCCACAA	

### Chromatin immunoprecipitation

This procedure is modified based on a published protocol [88]. For each genotype, approximately 800 eye-antennal discs were dissected. The samples were cross-linked with 1% formaldehyde in dissection buffer for 10min at room temperature. To quench cross-linking, glycine was added to 125mM final concentration, and the samples were incubated for 5min. The discs were homogenized and sonicated in a Bioruptor machine for 13min (high frequency; 30 sec ON/30 sec OFF). The chromatin was pre-cleared with pre-washed Dynabeads Protein G (Life Technologies) for 1hr at 4°C on a nutator. The pre-cleared chromatin was split into 2 tubes (1ml/tube), and another 20ul (2%) was saved as input and stored at -20°C. 5ug Anti-GFP antibody (Ab290) or an equal amount of normal rabbit IgG were added to either tube, followed by overnight incubation at 4°C. Beads were added to both tubes, and the samples were incubated for 2 hours at 4°C on a nutator. Beads was briefly rinsed with wash buffer I (50mM K-HEPES, pH7.8, 140mM NaCl, 1mM EDTA, 1mM EGTA, 1% Triton X-100, 0.1% Na-deoxycholate, 0.1% SDS), and washed 1X with wash buffer I, 1X with wash buffer II (the same as buffer I, except that NaCl is 500mM), 1X with wash buffer III (250mM LiCl, 0.5% Igepal CA-630, 0.5% Na-deoxycholate, 1XTE), 2X with the TE buffer, at 4°C, 5min/each wash. The chromatin was eluted 2X with pre-warmed elution buffer (1% SDS, 100mM NaHCO_3_). For each elution, beads were incubated in 100ul solution for 10min at 65°C, with gentle vortexing every 2–3 min. To reverse cross-link, 5M NaCl was added to each tube, followed by overnight incubation at 65°C. The ChIP-ed DNA was treated with RNase and proteinase K, and extracted by PCR purification columns (Qiagen). The purified DNA was tested for enrichment of DNA fragments by qPCR. For each target gene, up to 150bp amplicons were selected every ~300bp in the first 2kb region upstream of the coding region (Table 3). A primer pair covering T13 motif within the *bab2* LAE (leg and antennal enhancer) was used as a positive control for ChIP-qPCR analysis [39]. The M1 motif upstream of the *rn*-positive Or82 promoter was used as a negative control.

**Table 3:** ChIP-qPCR primers.

Primer Name	Sequence
Bab2_ChIP_T13_F	TATTTGCGTGGAGCCTTC
Bab2_ChIP_T13_R	TAACGATTGCCGCGATTT
Or82a_ChIP_M1_F	CACAGTACATACAGCCATACAG
Or82a_ChIP_M1_R	CGCTTCCTTCTGCTTGTT
B2_ChIP_399_F	CCCTCAAAGATAACGAACACG
B2_ChIP_399_R	CGAACTACAACCGCACAAA
B2_ChIP_728_F	TGAGTTTCAAGCTGCCATAA
B2_ChIP_728_R	TTGTACAGGAATGACAGAACAT
B2_ChIP_1588_F	CATGGTTTATTCAGAGGCAATAC
B2_ChIP_1588_R	CCAAGCATTTACGACCTGA

B1 and B2 stand for Bar-H1 and Bar-H2, respectively. T13 and M1 are the motif names [38,39]. The numbers correspond to the lengths between transcription start sites and the beginning of the amplicons.

### Statistical analysis

Statistical analysis of Bab2 expression levels, qPCR results, and neuron counts was by unpaired, two-tailed Student’s t test. Single factor ANOVA was used to analyze the number of Or47b neurons in *rn*/*Bar* double mutant analysis. Post-hoc, unpaired, two-tailed Student’s t tests were calculated after ANOVA. For all tests, * *p*< 0.1, ** *p*<0.01, *** *p*<0.001.

## Supporting Information

S1 Fig Summary of RNAseq analysis. (A) Venn diagram showing the numbers of genes misregulated in *rn* mutants in the three early stages T1 (3^rd^ instar larval), T2 (8hr APF), and T3 (40hr APF). APF: after puparium formation. (B) Summary of misregulated genes based on the directions.
(TIF)

S2 Fig Heatmap of olfactory receptor expression in the control and *rn* mutant adult antennae by RNAseq. Normalized expression of all olfactory receptors in the adult stage by DESeq2 is shown. Sequencing results of two biological replicates of paired-end reads per genotype were used as the input. Each transcription variant was treated individually.
(TIF)

S3 Fig Expression of PD genes in the antennal disc. (A) *ap* expression (red) visualized by the enhancer trap line *ap*^*rK568*^ remains inside the central fold (compared to Fig 3E and 3F). CF, central fold (dashed line). (B) Confocal images of the third instar larval antennal disc. Bab1 expression is visualized by staining the enhancer trap line *bab1*^*A128*^ with β-gal antibody. Bab2 is stained with its antibody (F. Laski). These two genes are partially redundant and overlapping in expression. Both genes show gradient expression and are confined within the boundary set by Dac (red). A single slice of the confocal image shows the boundary between Bab and Dac. Boxed area is shown on the right. Weak Bab2 slightly overlaps with Dac, while the overlapping between *bab1*^*A128*^ and Dac is not obvious, presumably due to the level of expression below the detectable range. (C) A side view of the antennal disc highlighting the central fold. Both Rn (green) and Bab (red) are present continuously throughout the central fold. White arrows point to the central fold. (D) Cartoon schematic showing the rings of the antennal disc as viewed from the side (also see Figs 3A and 5A).
(TIF)

S4 Fig Expression of GFP in the mid-pupal antennal lobe driven by *Bar* or *bab1* GAL4. (A) A confocal Z-projection showing neuropil (magenta) and *Bar*-GAL4 UAS-Syt GFP (green). (B) As in (A) but with *bab1*-GAL4. Both images were taken from approximately 50 hr APF pupal brains. This data was incorporated into Table 1.
(TIF)

S5 Fig Expansion of Or47b in Bar overexpression. (A) Quantification of cell counts of Or47b neurons in (B). Flies that overexpress BarH1 with the *rn*^*89GAL4*^ driver show significant reductions in the numbers of Or47b neurons in both females (blue) and males (red). * *p* < 0.05, ** *p* < 0.01. (B) Antennal images of Or47b neurons in BarH1 overexpressing flies. Although the number of Or47b neurons is reduced, we detect ectopic neurons (arrowheads) in the anterior portion of the antenna, consistent with the phenotype seen in *rn* mutants.
(TIF)

S6 Fig Changes in sensilla identities in *rn* mutant as well as Bar and Ap overexpression. (A-D) Glomerular targets of ab5 ORNs (blue dashed lines) and at4 ORNs (yellow dashed lines are shown in wild type (A), *rn* mutants (C), BarH1, and Ap overexpression (B and D, respectively). The glomerular targets of at4 ORNs are expanded in all cases and the targets of ab5 ORNs are lost in all cases. (E-M) Analysis of OR expression in adult antennae also corroborates qPCR data (Figs 6D and 7D). ab7 sensilla (Or98a and Or67c) are downregulated in BarH1 overexpression (F) and (I) but are upregulated in Ap overexpression (G) and (J) compared to (E) and (H). Or49a, which pairs with Or67a in ab10 sensilla, is downregulated in both Bar and Ap overexpression (K-M).
(TIF)

S7 Fig TF expression in Bar overexpression. (A) and (B) Single slices of 3^rd^ instar larval discs showing the expression patterns of Rn, *ap* and Dac in control and Bar-overexpressing lines. The central fold is highlighted as a dashed line and is absent in Bar-overexpressing larvae. No change was detected in Rn or *ap* staining. (C) and (D) Z-stacks of antennal discs stained for Bab and Dac in control and Bar-overexpressing discs. Beyond the loss of the central fold, no change in Bab expression was detected.
(TIF)

S8 Fig Changes in TF expression in Ap overexpression. (A) and (B) Single slices of 3^rd^ instar larval discs showing the expression patterns of Rn, Bab and Bar in control and Ap overexpressing lines. Bar is expanded outside of the central fold (arrowheads) in larvae that overexpress Ap. No change was detected in Bab staining. Rn expression is lost in R(5) inside of the central fold (arrows). (C) Loss of Rn (arrowheads) inside of the central fold (dashed line) in Ap overexpressing larvae. (D) Single slice of the confocal image shown in Fig 7C. Limit of Bar expansion is defined by Dac expression. Rn-positive, Dac-negative cells that do not express (asterisks) or express low levels of Bar (crosses) can be seen in the enlarged area (boxed).
(TIF)

S9 Fig Effect of loss of *apterous* or *bar* on OR expression. (A) Quantitative RT-PCR analysis for ORs in control (*ey-*FLP FRT19A/FM6) and *bar* mutant clones (*ey*-FLP FRT19A/D(f)1 Bar FRT19A). No significant changes were detected for all ORs tested. (B) Quantitative RT-PCR analysis of *w*^*1118*^ and *ap* mutants (*nap1/ap*^*md544GAL4*^). The expression of *IR31a* (ac1), *Or85a* (ab2), and *Or56a* (ab6) were significantly reduced in *ap* mutants. All three ORN classes were also shown to be positive for *ap* expression (Table 1 and Fig 5A). * *p* < 0.05, ** *p* < 0.01.
(TIF)

S10 Fig Overexpression of constitutively active EGFR. (A) Quantitative RT-PCR analysis of OR genes in UAS-GFP; *rn*^*89GAL4*^ and UAS-lambda top 4.4/ *rn*^*89GAL4*^ flies. *Or67b* (ab9), *Or13a* (ai1), and *IR84a* (ac4) were downregulated and *Or67a* (ab10) was upregulated in EGFR overexpressing flies (* *p* < 0.05, ** *p* < 0.01). (B) Staining on the control UAS-GFP; *rn*^*89GAL4*^ third instar larval discs for Bar (red), *rn*^*89*^ (green) and Dac (blue). The central fold (dashed line) and R(2)-(4) are highlighted. (C) Staining as in (B) in flies overexpressing EGFR. Bar-positive region had expanded and the location of the central fold had changed. Bar and Dac domains became adjacent to each other. There is also significant repression of *rn* expression, consistent with previous reports of the function of EGFR signaling.
(TIF)

S11 Fig Expression of Gr5a and Gr43a in *rn* mutant legs. (Top) The sweet sensing Gr5a neuron (co-expressing Gr61a) is expanded in *rn* mutants. Control flies have four Gr5a neurons (left, white asterisks) in the 4^th^ and 5^th^ tarsal segments. In *rn* mutants an ectopic neuron is present (right, red asterisk). (Bottom) The bitter sensing Gr43a neurons are unchanged in *rn* mutants, suggesting the expansion of Gr61 (Fig 9) comes from 5b/4s instead of 5v sensilla.
(TIF)

S1 Table Number of Or47b neurons in *rn* and *Bar* mutants. Raw data shown in Fig 2A and 2B of the number of Or47b neurons in each antennaa. Single factor ANOVA statistics are displayed at the bottom.
(DOCX)

S1 Text Detailed explanation of models presented in Figs 5B, 6A and 7A. This text provides more detailed explanations of how the models in Figs 5B, 6A and 7A were generated.
(DOCX)
