# Supplementary figures and images for: A Functionally Conserved Gene Regulatory Network Module Governing Olfactory Neuron Diversity
Source: PLoS Genet. 2016 Jan 14;12(1):e1005780. doi: 10.1371/journal.pgen.1005780 (PMC4713227; doi:10.1371/journal.pgen.1005780)

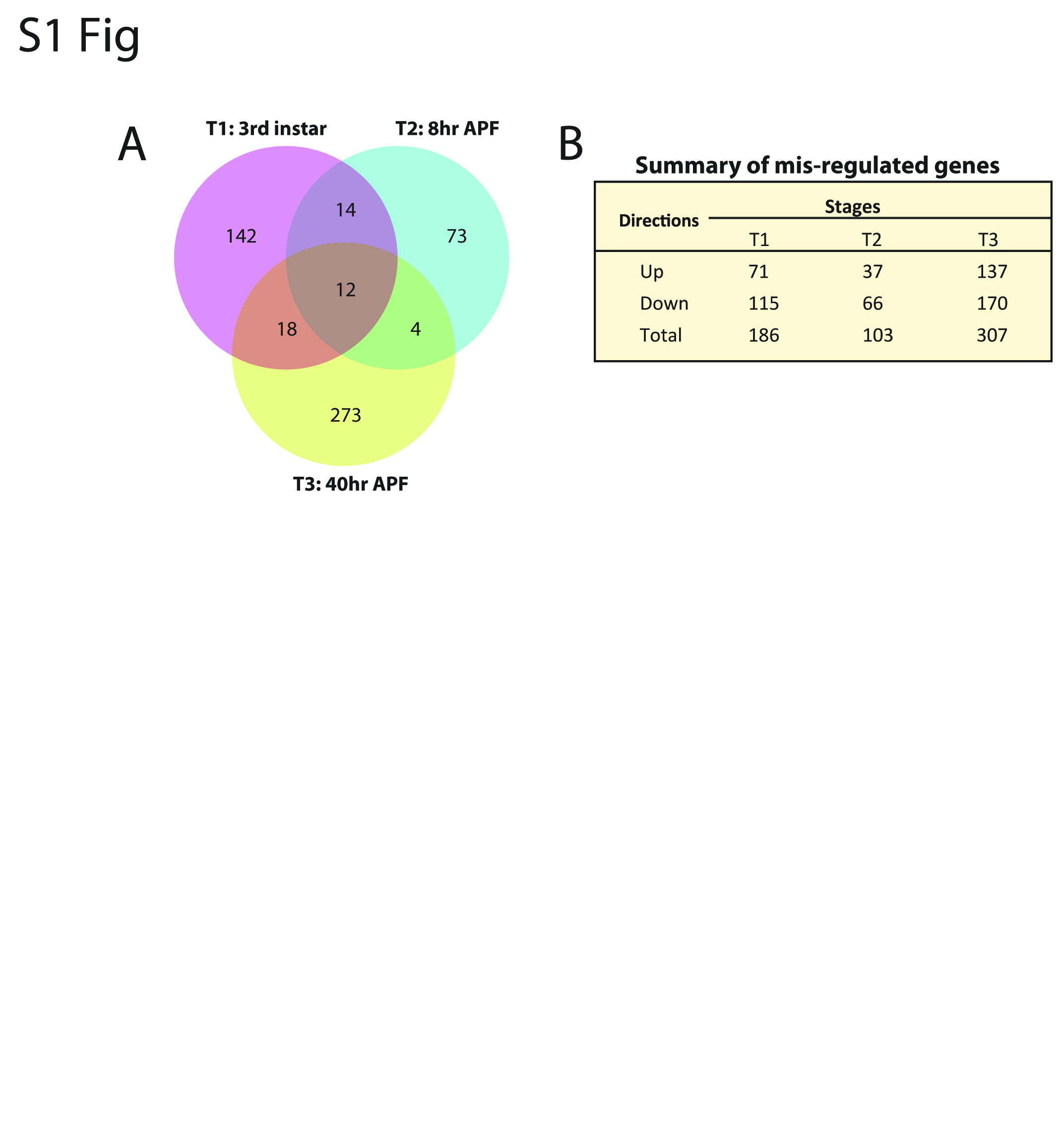

Supplement: S1 Fig — (A) Venn diagram showing the numbers of genes misregulated in rn mutants in the three early stages T1 (3rd instar larval), T2 (8hr APF), and T3 (40hr APF). APF: after puparium formation. (B) Summary of misregulated genes based on the directions. (TIF) [file pgen.1005780.s001.tif]

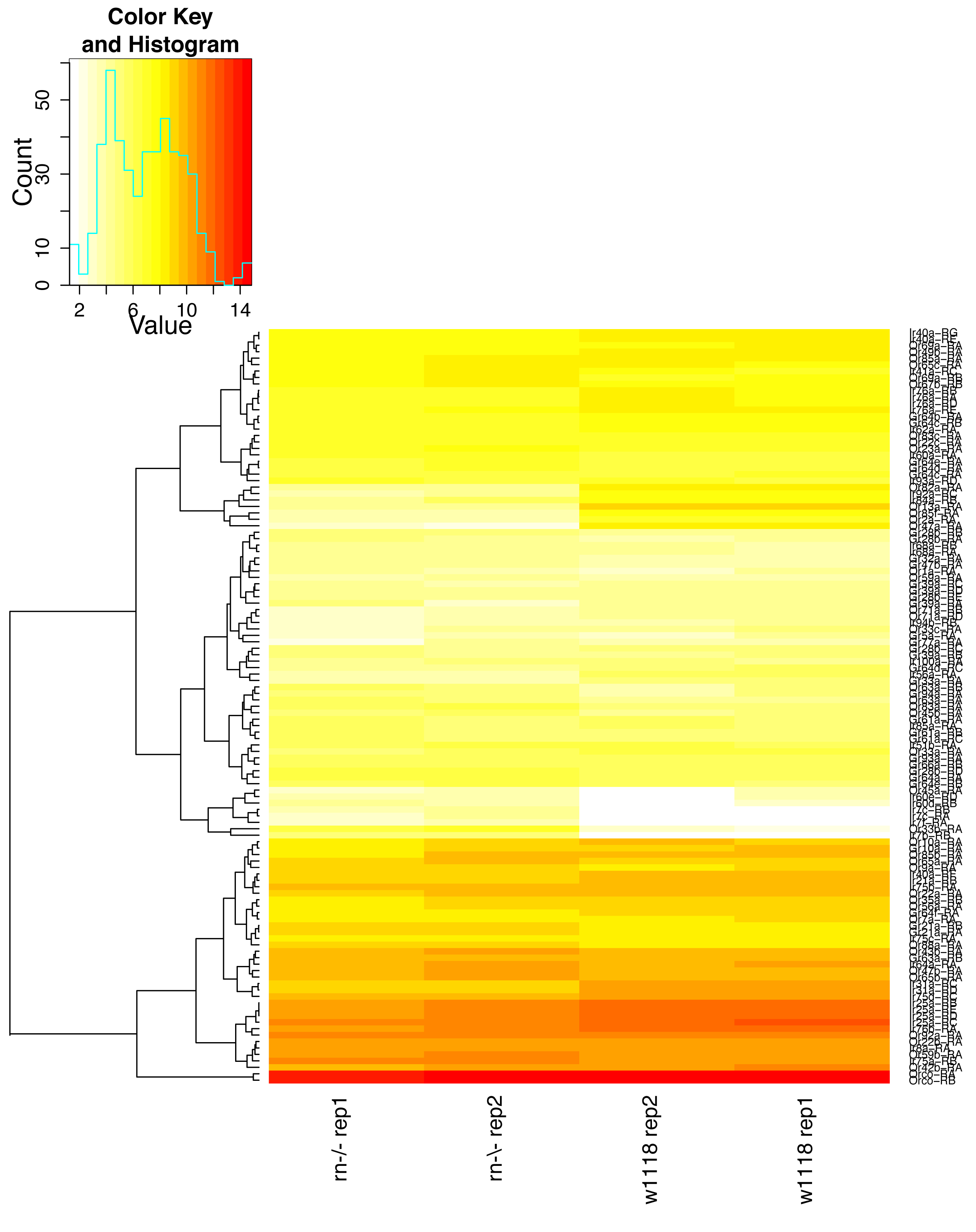

Supplement: S2 Fig — Normalized expression of all olfactory receptors in the adult stage by DESeq2 is shown. Sequencing results of two biological replicates of paired-end reads per genotype were used as the input. Each transcription variant was treated individually. (TIF) [file pgen.1005780.s002.tif]

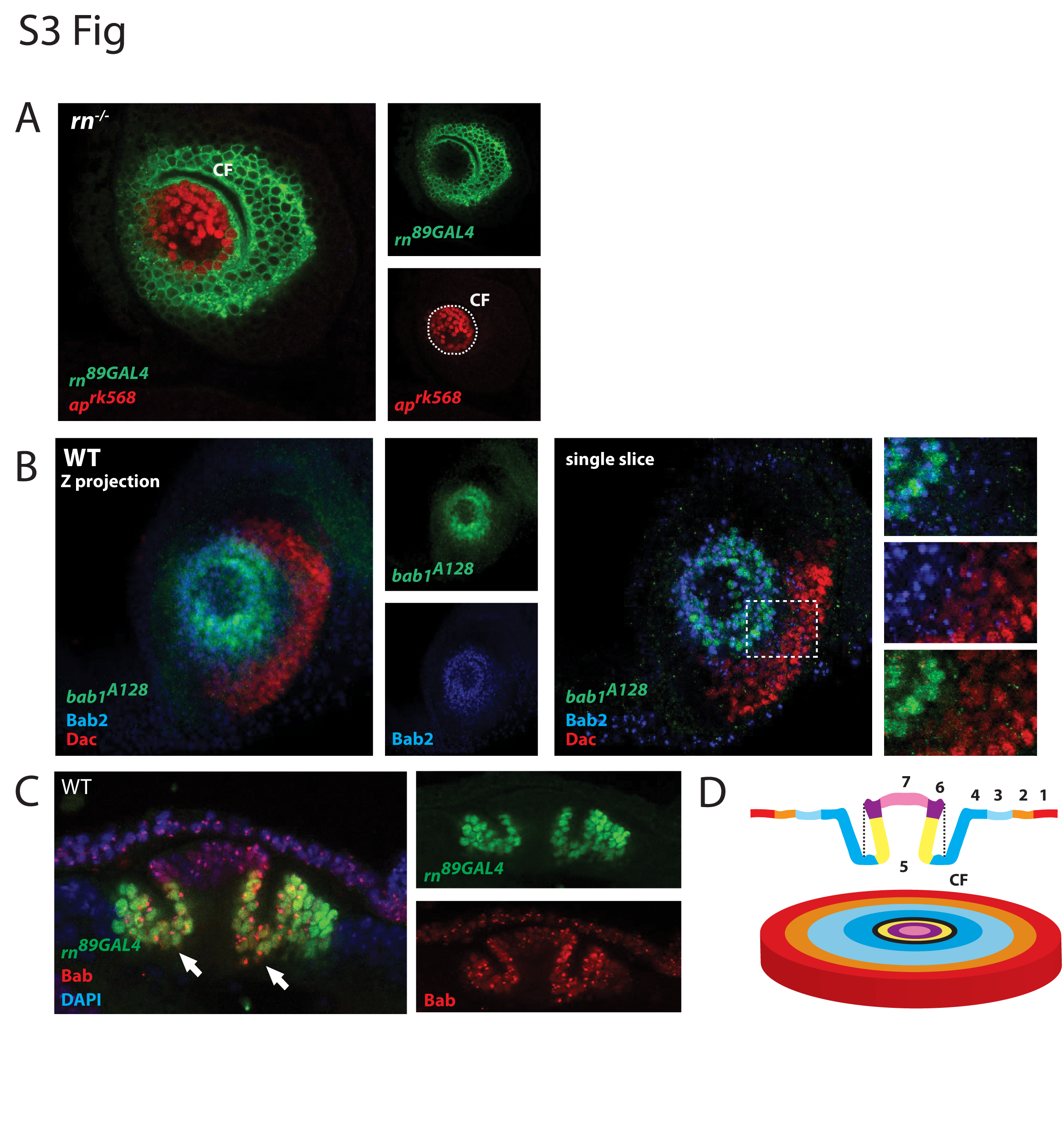

Supplement: S3 Fig — (A) ap expression (red) visualized by the enhancer trap line aprK568 remains inside the central fold (compared to Fig 3E and 3F). CF, central fold (dashed line). (B) Confocal images of the third instar larval antennal disc. Bab1 expression is visualized by staining the enhancer trap line bab1A128 with β-gal antibody. Bab2 is stained with its antibody (F. Laski). These two genes are partially redundant and overlapping in expression. Both genes show gradient expression and are confined within the boundary set by Dac (red). A single slice of the confocal image shows the boundary between Bab and Dac. Boxed area is shown on the right. Weak Bab2 slightly overlaps with Dac, while the overlapping between bab1A128 and Dac is not obvious, presumably due to the level of expression below the detectable range. (C) A side view of the antennal disc highlighting the central fold. Both Rn (green) and Bab (red) are present continuously throughout the central fold. White arrows point to the central fold. (D) Cartoon schematic showing the rings of the antennal disc as viewed from the side (also see Figs 3A and 5A). (TIF) [file pgen.1005780.s003.tif]

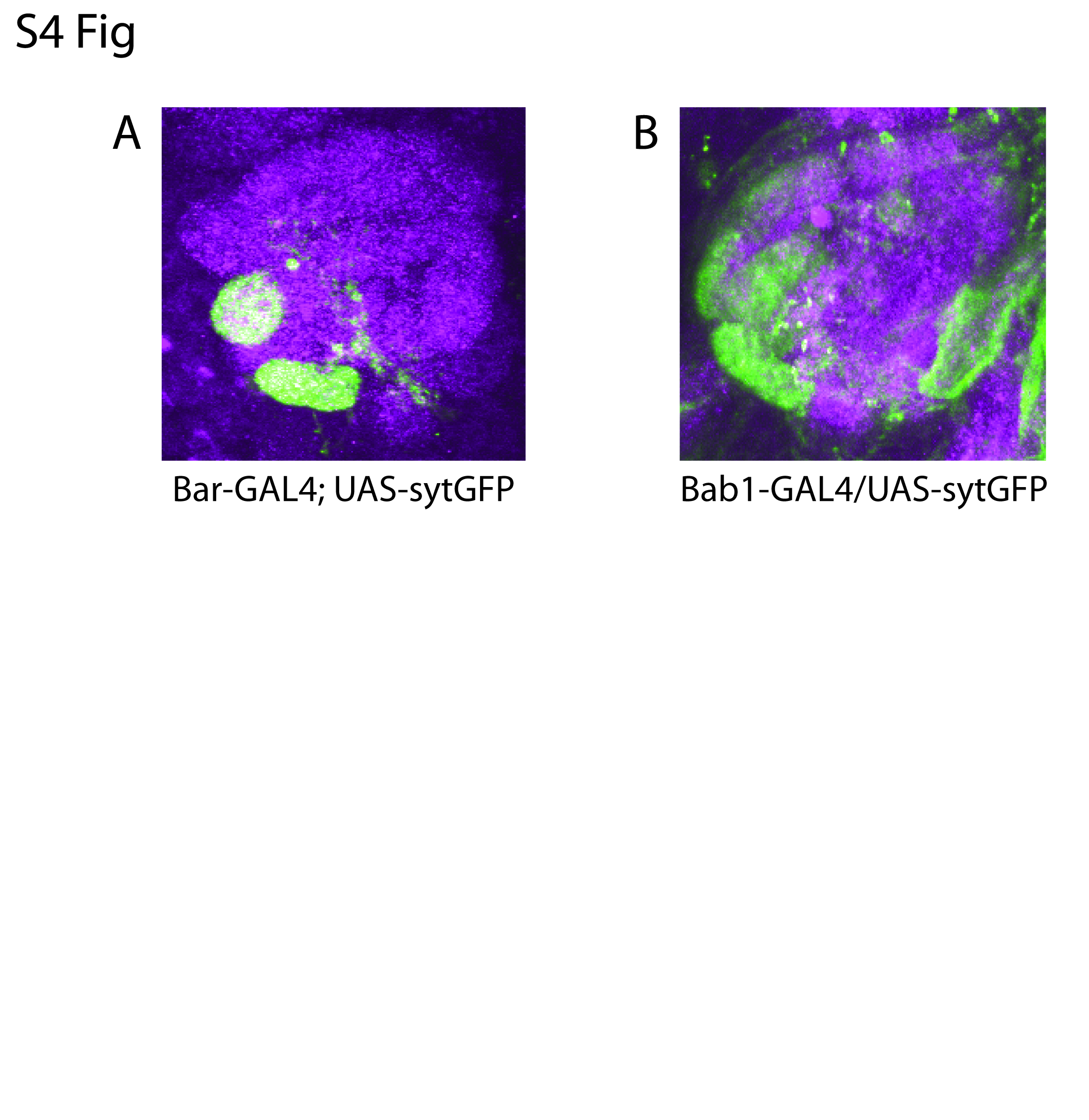

Supplement: S4 Fig — (A) A confocal Z-projection showing neuropil (magenta) and Bar-GAL4 UAS-Syt GFP (green). (B) As in (A) but with bab1-GAL4. Both images were taken from approximately 50 hr APF pupal brains. This data was incorporated into Table 1. (TIF) [file pgen.1005780.s004.tif]

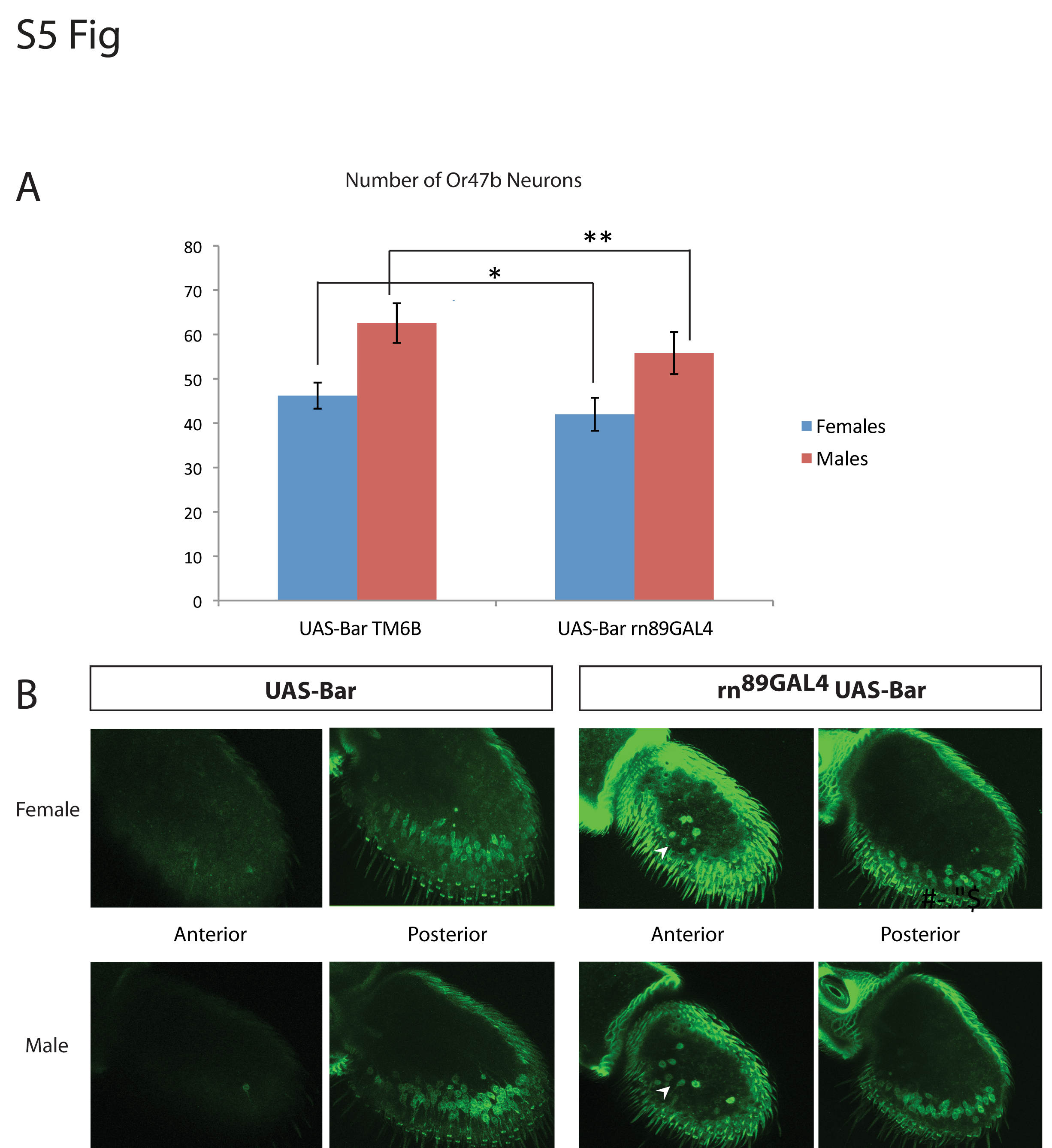

Supplement: S5 Fig — (A) Quantification of cell counts of Or47b neurons in (B). Flies that overexpress BarH1 with the rn89GAL4 driver show significant reductions in the numbers of Or47b neurons in both females (blue) and males (red). * p < 0.05, ** p < 0.01. (B) Antennal images of Or47b neurons in BarH1 overexpressing flies. Although the number of Or47b neurons is reduced, we detect ectopic neurons (arrowheads) in the anterior portion of the antenna, consistent with the phenotype seen in rn mutants. (TIF) [file pgen.1005780.s005.tif]

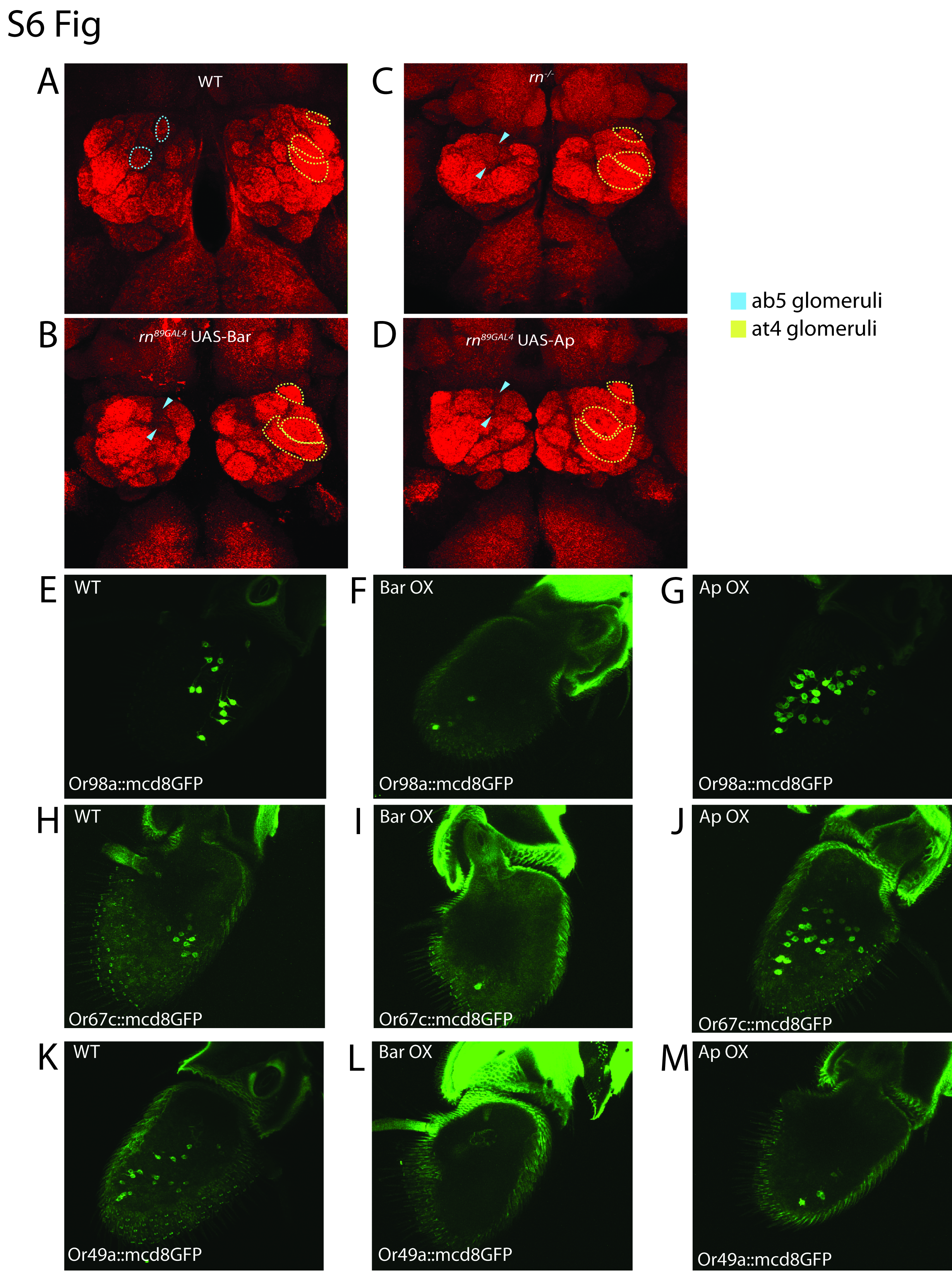

Supplement: S6 Fig — (A-D) Glomerular targets of ab5 ORNs (blue dashed lines) and at4 ORNs (yellow dashed lines are shown in wild type (A), rn mutants (C), BarH1, and Ap overexpression (B and D, respectively). The glomerular targets of at4 ORNs are expanded in all cases and the targets of ab5 ORNs are lost in all cases. (E-M) Analysis of OR expression in adult antennae also corroborates qPCR data (Figs 6D and 7D). ab7 sensilla (Or98a and Or67c) are downregulated in BarH1 overexpression (F) and (I) but are upregulated in Ap overexpression (G) and (J) compared to (E) and (H). Or49a, which pairs with Or67a in ab10 sensilla, is downregulated in both Bar and Ap overexpression (K-M). (TIF) [file pgen.1005780.s006.tif]

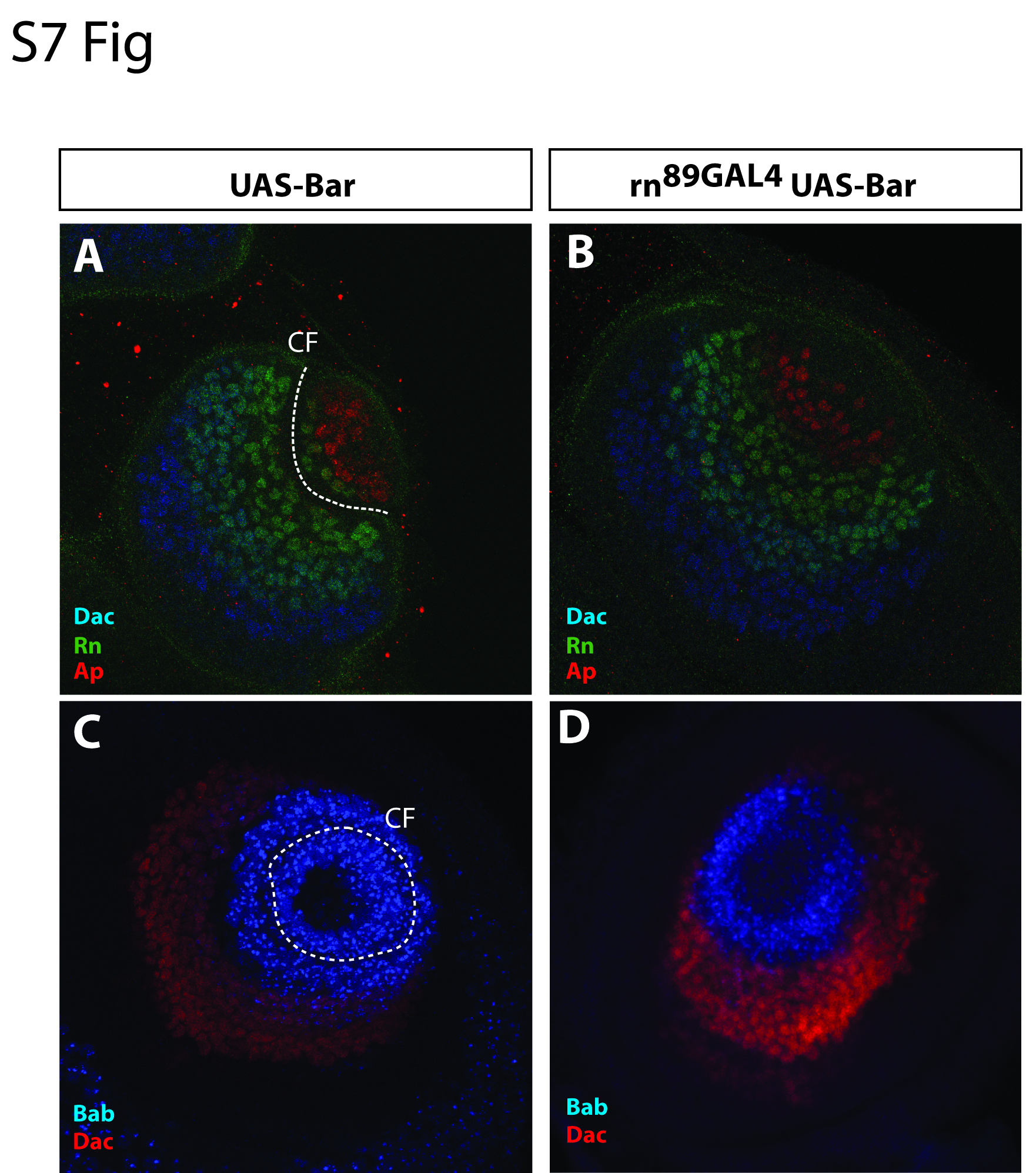

Supplement: S7 Fig — (A) and (B) Single slices of 3rd instar larval discs showing the expression patterns of Rn, ap and Dac in control and Bar-overexpressing lines. The central fold is highlighted as a dashed line and is absent in Bar-overexpressing larvae. No change was detected in Rn or ap staining. (C) and (D) Z-stacks of antennal discs stained for Bab and Dac in control and Bar-overexpressing discs. Beyond the loss of the central fold, no change in Bab expression was detected. (TIF) [file pgen.1005780.s007.tif]

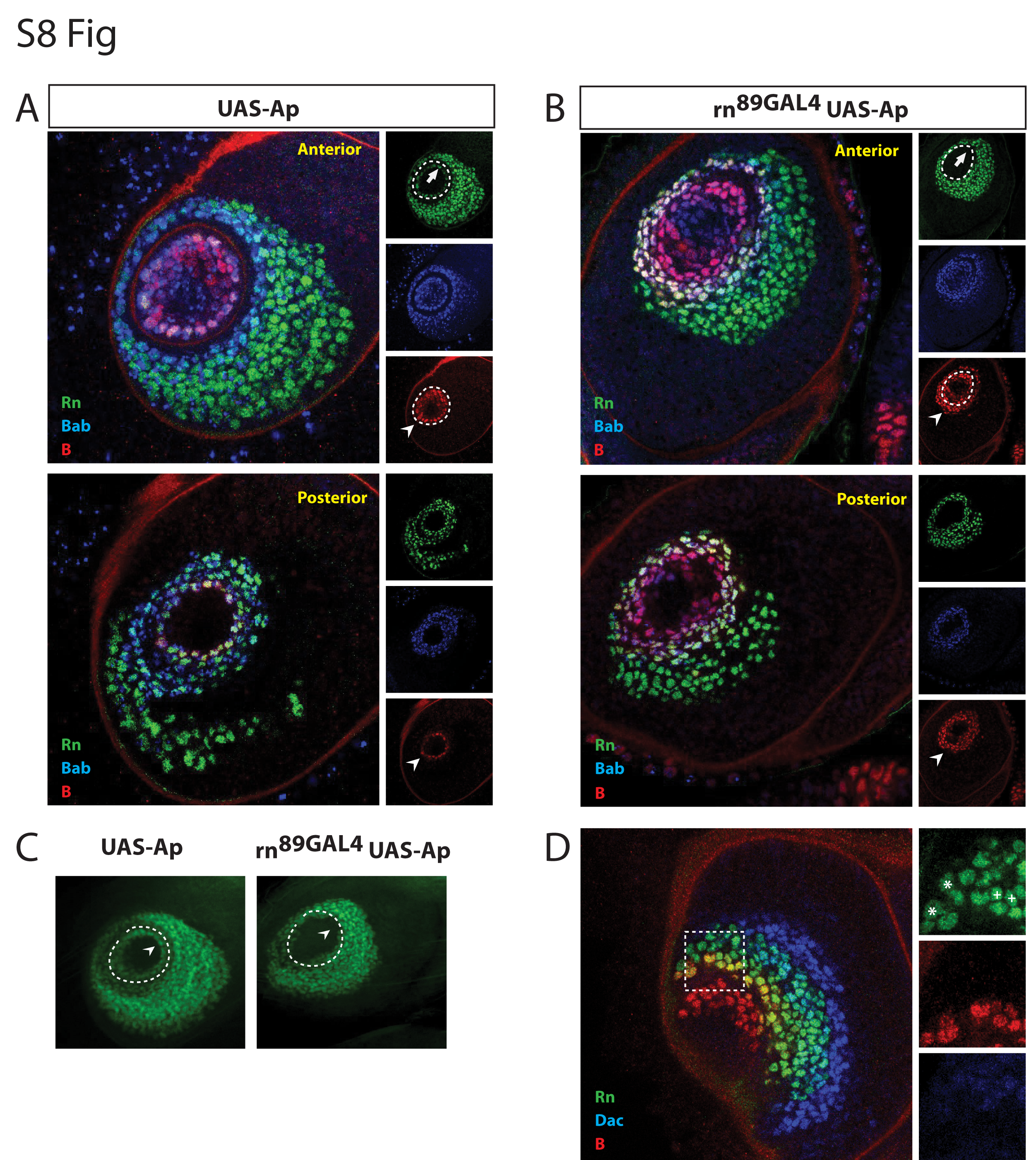

Supplement: S8 Fig — (A) and (B) Single slices of 3rd instar larval discs showing the expression patterns of Rn, Bab and Bar in control and Ap overexpressing lines. Bar is expanded outside of the central fold (arrowheads) in larvae that overexpress Ap. No change was detected in Bab staining. Rn expression is lost in R(5) inside of the central fold (arrows). (C) Loss of Rn (arrowheads) inside of the central fold (dashed line) in Ap overexpressing larvae. (D) Single slice of the confocal image shown in Fig 7C. Limit of Bar expansion is defined by Dac expression. Rn-positive, Dac-negative cells that do not express (asterisks) or express low levels of Bar (crosses) can be seen in the enlarged area (boxed). (TIF) [file pgen.1005780.s008.tif]

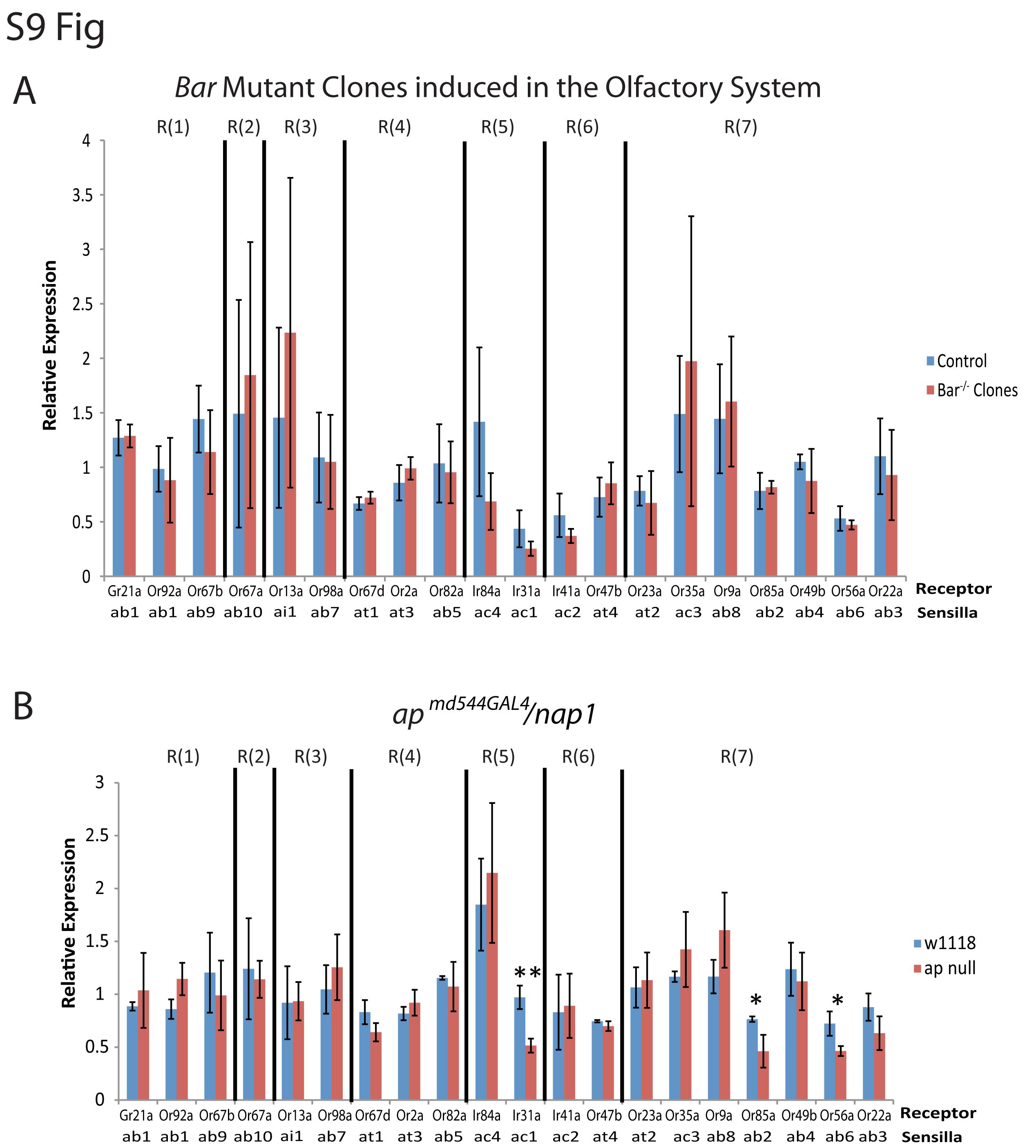

Supplement: S9 Fig — (A) Quantitative RT-PCR analysis for ORs in control (ey-FLP FRT19A/FM6) and bar mutant clones (ey-FLP FRT19A/D(f)1 Bar FRT19A). No significant changes were detected for all ORs tested. (B) Quantitative RT-PCR analysis of w1118 and ap mutants (nap1/apmd544GAL4). The expression of IR31a (ac1), Or85a (ab2), and Or56a (ab6) were significantly reduced in ap mutants. All three ORN classes were also shown to be positive for ap expression (Table 1 and Fig 5A). * p < 0.05, ** p < 0.01. (TIF) [file pgen.1005780.s009.tif]

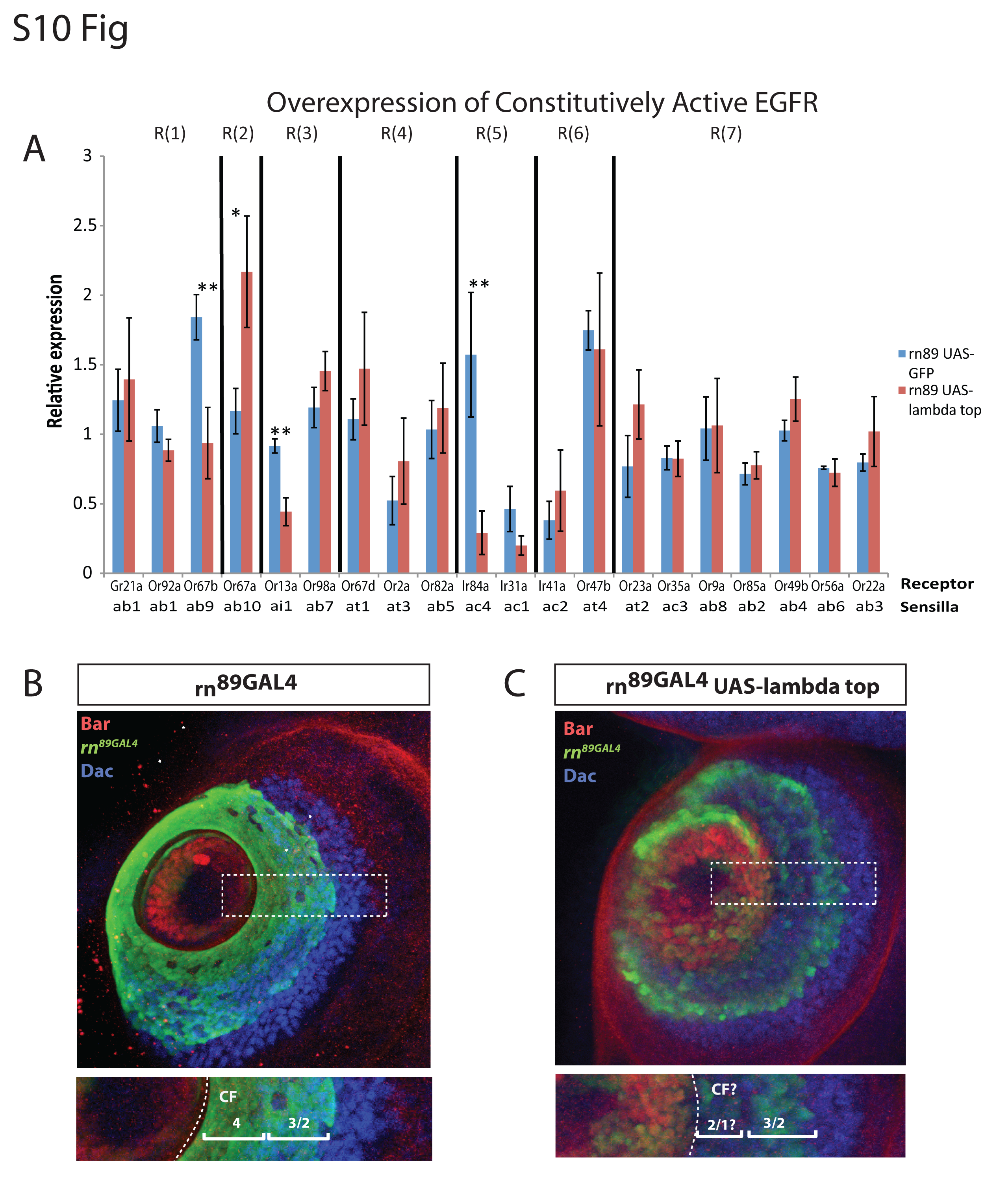

Supplement: S10 Fig — (A) Quantitative RT-PCR analysis of OR genes in UAS-GFP; rn89GAL4 and UAS-lambda top 4.4/ rn89GAL4 flies. Or67b (ab9), Or13a (ai1), and IR84a (ac4) were downregulated and Or67a (ab10) was upregulated in EGFR overexpressing flies (* p < 0.05, ** p < 0.01). (B) Staining on the control UAS-GFP; rn89GAL4 third instar larval discs for Bar (red), rn89 (green) and Dac (blue). The central fold (dashed line) and R(2)-(4) are highlighted. (C) Staining as in (B) in flies overexpressing EGFR. Bar-positive region had expanded and the location of the central fold had changed. Bar and Dac domains became adjacent to each other. There is also significant repression of rn expression, consistent with previous reports of the function of EGFR signaling. (TIF) [file pgen.1005780.s010.tif]

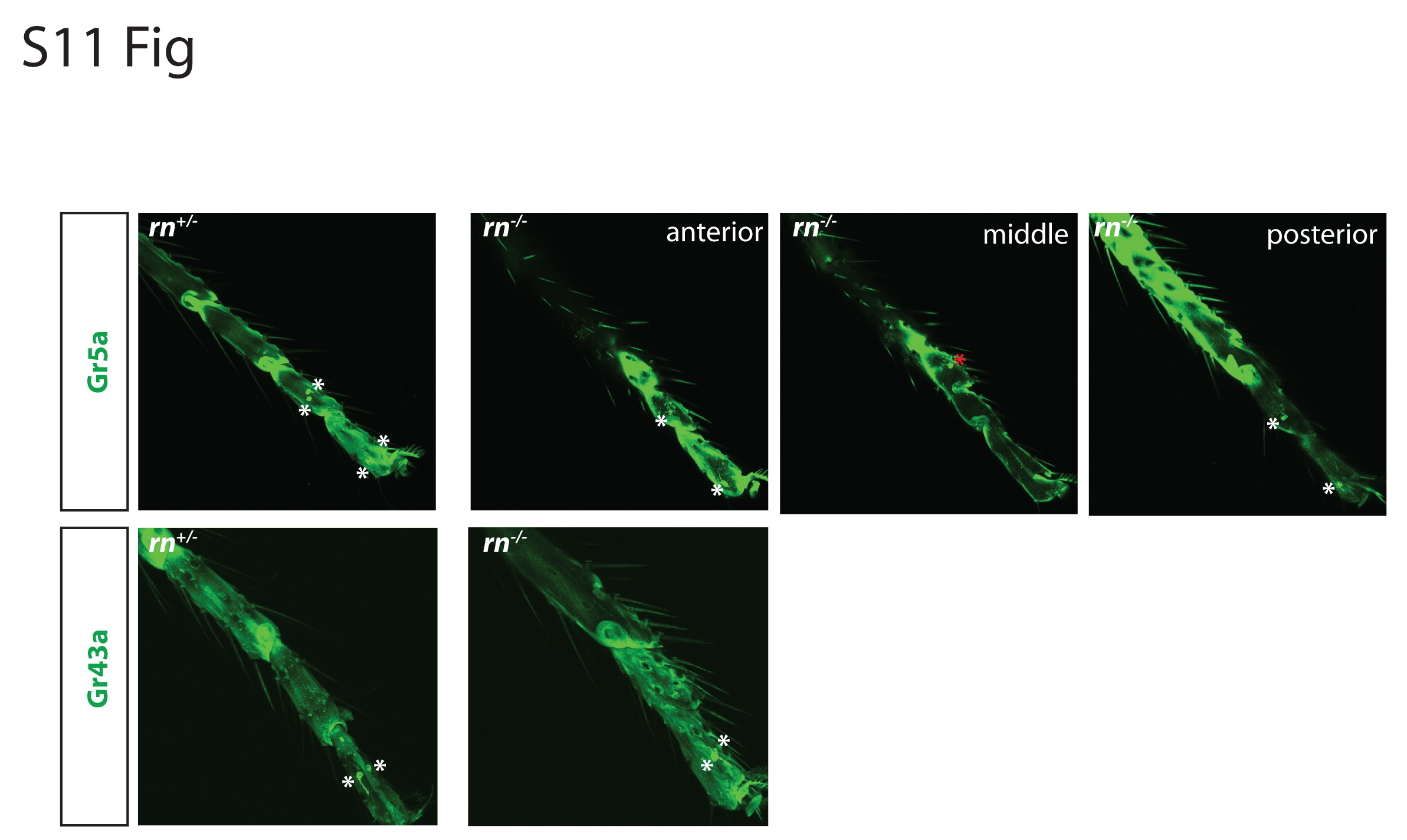

Supplement: S11 Fig — (Top) The sweet sensing Gr5a neuron (co-expressing Gr61a) is expanded in rn mutants. Control flies have four Gr5a neurons (left, white asterisks) in the 4th and 5th tarsal segments. In rn mutants an ectopic neuron is present (right, red asterisk). (Bottom) The bitter sensing Gr43a neurons are unchanged in rn mutants, suggesting the expansion of Gr61 (Fig 9) comes from 5b/4s instead of 5v sensilla. (TIF) [file pgen.1005780.s011.tif]
