# Supplementary material for: A Functionally Conserved Gene Regulatory Network Module Governing Olfactory Neuron Diversity
Source: PLoS Genet. 2016 Jan 14;12(1):e1005780. doi: 10.1371/journal.pgen.1005780 (PMC4713227; doi:10.1371/journal.pgen.1005780)
Supplement: S1 Table — Raw data shown in Fig 2A and 2B of the number of Or47b neurons in each antennaa. Single factor ANOVA statistics are displayed at the bottom. (DOCX) [file pgen.1005780.s012.docx]

**S1 Table. Number of Or47b neurons in *rn* and *Bar* mutants.**

| Genotype | Bar +/-; rn +/- | Bar +/-; rn -/- | Bar-/-; rn-/- |
| --- | --- | --- | --- |
|  | 54 | 88 | 67 |
|  | 61 | 87 | 80 |
|  | 56 | 102 | 82 |
|  | 66 | 101 | 82 |
|  | 65 | 91 | 94 |
|  | 57 | 92 | 88 |
|  | 54 | 93 | 82 |
|  | 55 | 83 | 97 |
|  | 54 | 91 | 77 |
|  | 46 | 91 | 64 |
|  |  | 88 | 79 |
|  |  | 110 | 84 |
|  |  | 91 | 103 |
|  |  | 95 | 81 |
|  |  | 104 | 64 |
| Average | 56.8 | 93.8 | 81.6 |
| Anova: Single Factor | *P-value* | *F crit* | *df* |
|  | 1.01E-11 | 3.251924 | 39 |
