# Supplementary material for: A Functionally Conserved Gene Regulatory Network Module Governing Olfactory Neuron Diversity
Source: PLoS Genet. 2016 Jan 14;12(1):e1005780. doi: 10.1371/journal.pgen.1005780 (PMC4713227; doi:10.1371/journal.pgen.1005780)
Supplement: S1 Text — This text provides more detailed explanations of how the models in Figs 5B, 6A and 7A were generated. (DOCX) [file pgen.1005780.s013.docx]

**S1 Text. Detailed explanation of models presented in Figs 5B, 6A, and 7A.**

**Explanation for the model in Fig 5B**

First, we wanted to use our model to explain the *rn* mutant phenotype. In the *rn* mutant imaginal disc, *Bar* is de-repressed and expression expands outside of the central fold, but this expansion respects the boundary between R(3) and R(4) (Figs 1C and 3A). Consequently, the new proximal limit of Bar is abutted by the distal limit of Dac. Another prominent phenotype is the reduction of Bab expression due to the lack of Rn activation, resulting in a shallower concentration gradient (Fig 1D, E). The combined effects, along with the loss of Rn, alter the combinatorial code of transcription factor expression for each ring. Particularly, R(2) and the proximal part of R(3) become Dac only, converting the precursor potentials to R(1) potentials. We found that this conversion in precursor fates can explain the loss of ai1, ab7 and ab10 subtypes and the expansion of ab1 and ab9 observed in *rn* mutants (1). Although we do not have markers to show the origins of sensilla subtype conversions, we suspect that ab7 and ab10 may be converted to ab9 within the small basiconic sub-lineage, and ai1 might be converted to ab1. The precursors in the distal part of R(3) may die because of an invalid code.

Further, the code in R(5) is changed to the same one as in R(6), particularly because the decrease in Bab brings its level in R(5) closer to the level in R(6), while R(6) itself is Rn-independent and thus unaffected. This change leads to the loss of R(5) and expansion of R(6). Meanwhile, the ectopic Bar in R(4) changes its code to Bab and Bar. We could not, however, detect expansion of *ap* in R(4) (S3A Fig). It is possible that in this context Ap is not required to protect Bar from being repressed by Rn because Rn is not present. Therefore, Bab and Bar expression in R(4) in *rn* mutants is equal to the code in R(6), and is sufficient to specify R(6) fates. Consistent with this argument, Ap function is not required for the specification of ac2 or at4 sensilla subtypes (S9B Fig), which are generated from R(6) according to our model. Based on this reasoning, it would appear that the major function of Ap is to protect Bar from being repressed by Rn in the quadruple positive zone R(5) consistent with previous reports (2–4). This function does not occur at the transcriptional level, but is perhaps mediated by formation of a complex with Bar and/or Rn to counteract Rn’s repressive function. Consistently, Ap has been shown to interact with Bar in a protein complex (2,4). Therefore, our model can explain the loss of at1, at3, ab5, ac4 and ac1 sensilla subtypes from R(4) and R(5), and the expansion of ac2 and at4 normally specified in R(6). Again, we speculate that the conversions occur within sensilla type lineages: at1 and at3 are converted to at4; ac1 and ac4 are converted to ac2; ab5 might be lost due to an incompatible Bab level. Taken together, our model provides an explanation for lineage-specific conversions in sensilla subtypes found in *rn* mutants by changes in the network code.

**Explanation for the model in Fig 6A**

We next wanted to use our model to explain the changes in sensilla subtype composition in Bar-overexpression flies. As Ap is not activated in ectopic domains in response to Bar overexpression (S7B Fig), precursors in R(4) acquire the new code of Rn, Bab, and Bar. Despite the absence of Ap, this code could be sufficient to confer the fates normally specified in R(5), leading to sensilla subtype conversions from at1, at3 and ab5 to ac4 and ac1. The overexpression of Bar in R(5) may inhibit Rn expression in this ring similar to the context-dependent repression of Rn by Ap in this ring. It is unclear whether this repression occurs due to the disappearance of the central fold (Fig 6C). Nevertheless, it would appear that the precursors in R(5) are converted to R(6), causing the endogenous ac4 and ac1 to be converted to ac2 and at4. Accordingly, we saw increases in the transcription of receptors in ac2 and expansion of ORNs in at4 subtypes (Fig 6D, S5 Fig). Due to the large numbers of at1, at3 and ab5 being converted to ac4 and ac1, the overall numbers of ac4 and ac1 may be still increased despite the conversion of the endogenous population. Indeed, we found that receptors expressed by these two sensilla are up-regulated in Bar overexpression (Fig 6D). We also found that a number of sensilla subtypes from R(7) are increased (Fig 6D). Although we do not have a satisfying explanation for it at the moment, we suspect that the ectopic sensilla may originate from the Bab-positive R(3), in which this new code somehow is competent to induce the fates normally specified in the center of the disc. Lastly, ab10 from R(2) may be lost due to the toxicity of the new code in this ring, which is consistent with the down-regulation of the tested receptor in ab10.

**Explanation for the model in Fig 7A**

When we used *rn^89GAL4^* to drive UAS-ap, we found Rn expression in R(5) is lost (S8A-C Fig). Due to the code change, R(6) is expanded into R(5), leading to sensilla subtype conversions from ac4 and ac1 to ac2 and at4. In addition, we found Bar is expanded close to the R(3) and R(4) boundary (Fig 7C, S8 Fig). As a result, the cells in the proximal portion of R(4) express Rn, Bab and Ap (S8B, D Fig). We think that this subpopulation of cells may be converted to fates in R(7) in this line, because: 1) qPCR results showed that some sensilla subtypes from R(7) are increased; 2) all sensilla subtypes from R(4) are significantly reduced; 3) the new code is similar to that in R(7), except that it contains Rn (Fig 7A). One explanation for why the presence of Rn does not interfere with the rest factors specifying R(7) fates is that Ap may interact with Rn to counteract its function (2,4). On the other hand, the cells in the distal half of R(4) become quadruple positive for factors normally found in R(5), resulting in sensilla subtype conversions from at1, at3 and ab5 to ac4 and ac1. We noticed that ac4 and ac1 are changed in opposite directions (Fig 7D). This is likely caused by endogenous cells in R(5) being further converted to the fates in R(6) as mentioned earlier. The differences in cell numbers that are converted during this relay may account for the different phenotypes for these two sensilla subtypes. Furthermore, sensilla subtypes from R(6) are increased and the corresponding glomeruli appear bigger in the overexpression line (S6A and 6D Fig).

We have shown that Dac levels are reduced in R(2) and R(3), and we deduced that Bab expression should be elevated, despite our inability to detect changes in Bab expression. Based on our findings about the function of Bab concentration gradients (see a later section in the main text), we think that this hypothetical de-repression of Bab may cause ai1, which requires lower levels of Bab, to be converted to ab7, requiring higher levels of Bab, in R(3). However, it is unclear why the overexpression of Ap in this domain does not affect ab7 specification. Finally, ab10 sensilla are dramatically reduced in Ap overexpression (Fig 7D). This may be due to some toxic effect of this non-existing code, such as the blocking of Rn function by Ap leaving only low levels of Dac, which is insufficient to specify any fate. Taken together, the proposed model can by and large explain the ORN phenotypes seen in Ap overexpression flies.

An interesting phenomenon is that, even though Bar expansion is a critical contributor to the *rn* mutant phenotype, and indeed many sensilla subtypes in the Ap/Bar overexpression lines show similar changes to those in *rn* mutants, the causes are very different in these situations based on our model. Because the intercalated patterns of these transcription factors, changes to one factor often lead to a domino effect involving many neighboring domains. In this sense, the similar phenotypes described above are essentially incidental and occur through distinct mechanisms.

**References**

1. Li Q, Ha TSS, Okuwa S, Wang Y, Wang Q, Millard SSS, et al. Combinatorial Rules of Precursor Specification Underlying Olfactory Neuron Diversity. Curr Biol [Internet]. Elsevier Ltd; 2013 Nov [cited 2013 Nov 25];1–10. Available from: http://linkinghub.elsevier.com/retrieve/pii/S0960982213013286

2. Natori K, Tajiri R, Furukawa S, Kojima T. Progressive tarsal patterning in the Drosophila by temporally dynamic regulation of transcription factor genes. Dev Biol [Internet]. Elsevier Inc.; 2012;361(2):450–62. Available from: http://dx.doi.org/10.1016/j.ydbio.2011.10.031

3. Pueyo JI, Galindo MI, Bishop S a, Couso JP. Proximal-distal leg development in Drosophila requires the apterous gene and the Lim1 homologue dlim1. Development. 2000;127:5391–402.

4. Pueyo JI, Couso JP. Chip-mediated partnerships of the homeodomain proteins Bar and Aristaless with the LIM-HOM proteins Apterous and Lim1 regulate distal leg development. Development. 2004;131:3107–20.
